# Selecting reasonable soil moisture-maintaining measures to improve the soil physicochemical properties and achieve high yield and quality of purple garlic in the China Hexi Corridor oasis agricultural area

**DOI:** 10.3389/fpls.2024.1447469

**Published:** 2024-09-12

**Authors:** Xiaofan Pan, Hengjia Zhang, Haoliang Deng, Shouchao Yu, Chenli Zhou, Fuqiang Li

**Affiliations:** ^1^ State Key Laboratory of Aridland Crop Science, College of Water Conservancy and Hydropower Engineering, Gansu Agricultural University, Lanzhou, China; ^2^ College of Agriculture and Biology, Liaocheng University, Liaocheng, China; ^3^ College of Civil Engineering, Hexi University, Zhangye, China

**Keywords:** soil physicochemical properties, purple garlic, yield, quality, oxo-biodegradable plastic film, Hexi Corridor oasis

## Abstract

Agricultural plastic film, as an important agricultural production material in the China Hexi Corridor oasis agricultural area, is widely used in the intensive production process of purple garlic, which plays an important role in increasing yield, improving quality, ensuring supply, etc. However, the difference in decomposition characteristics between ordinary plastic film and degradable plastic film may affect soil moisture and temperature, thereby affecting soil biochemical properties. Therefore, we conducted a study to solve this problem. Specifically, in the Minle area of the Hexi Corridor, we selected 10 moisture-maintaining measures of ordinary transparent plastic film, transparent oxo-biodegradable plastic film (50-, 80-, and 110-day induction period), ordinary black plastic film, black oxo-biodegradable plastic film (50-, 80-, and 110-day induction period), wheat straw, and aubergine-super absorbent polymers and used the traditional open field without super absorbent polymers as a control. To analyze the effects of different moisture-maintaining measures on soil quality, garlic yield and quality, and water-fertilizer productivity in purple garlic farmland, and conduct a comprehensive evaluation of moisture-maintaining measures using principal component analysis. The results showed that all the moisture-maintaining measures could increase garlic yield, improve bulb quality and water–fertilizer productivity, improve the soil hydrothermal conditions, maintain soil fertility, increase the microbial quantity, and improve enzyme activity. Overall, transparent plastic film mulching was superior to black plastic film mulching, straw mulching, and A-SAP, with 110-day transparent oxo-biodegradable plastic film mulching being the most effective, and was not significantly different from the ordinary transparent plastic film. Compared with other moisture-maintaining measures, the yield, water productivity, irrigation water productivity, and nitrogen fertilizer partial factor productivity of purple garlic were significantly increased by 13.33% to 119.77%, 13.81% to 126.77%, 13.41% to 119.95%, and 13.33% to 119.76%, respectively. Meanwhile, the contents of allicin, soluble sugar, soluble protein, crude fiber, and amino acid content were increased by 1.44% to 14.66%, 4.64% to 36.46%, 0.38% to 28.27%, 1.89% to 26.29%, and 0.38% to 3.74%, and, due to the prolongation of oxo-biodegradable plastic film induction period, the soil microbial community changes from “fungi type” to “bacterium type,” reducing the occurrence of soil diseases and improving soil quality. On the basis of the Technique for Order Preference by Similarity to an Ideal Solution (TOPSIS) method, the soil quality was evaluated, and the yield, quality, and water productivity of garlic were comprehensively evaluated under each moisture-maintaining measure using principal component analysis. It was determined that the best soil quality and better bulb quality as well as higher garlic yield and water productivity were obtained when using the 110-day induction period transparent oxo-biodegradable plastic film. It can be used as a more reasonable moisture-maintaining measure and technical reference for the purple garlic industry in the China Hexi Corridor oasis agricultural area, which can ensure the improvement of quality and stabilization of yield and also solve the risk of environmental pollution caused by plastic film mulching at the source.

## 1 Introduction

Dryland accounts for about 52.5% of the total arable land in China. The sustainable and stable development of dryland agriculture is related to national food security, effective supply of agricultural products, and sustained income increase for farmers in dryland areas (Lv and Ji, 2013). However, dryland agriculture is the most sensitive to climate change, and its development is deeply affected and constrained by temperature and precipitation (Oo et al., 2020). The plastic film–mulching technology was introduced to China in 1978 because of its important functions such as increasing temperature (Zhang et al., 2019), maintaining moisture (Yang et al., 2018), inhibiting inter-plant evaporation (Li et al., 2021a), weeding (Wang et al., 2020), preventing soil erosion (Lin et al., 2010), improving soil pH (Wang et al., 2017b), reducing soil nitrogen loss (Zhu et al., 2022), increasing microbial diversity and richness (Dong et al., 2017), improving crop resource utilization efficiency (Quan et al., 2022), and increasing crop yields (Fu et al., 2021). It is widely used in dryland agricultural areas and has also become one of the most important material production means in dryland agricultural production. China is the world’s largest consumer of agricultural plastic film, accounting for 75% of the global total (Petrescu-Mag et al., 2020), and the use of plastic film has reached 1.357 million t in 2020, with a total coverage area of 17.387 million hm^2^, accounting for about 12.89% of the total sown area of crops (China Rural Statistical Yearbook, 2021) and covering more than 40 types of crops, including grains, melons, fruits, vegetables, flowers, and grasses (Zhao et al., 2023), and it will remain at a high level for a long time. By 2025, projections estimate that China’s consumption of plastic film will soar to 2.28 million tons, covering an area spanning 23.4 million hm^2^ (Qi et al., 2020). Nonetheless, the prevalent agricultural plastic film materials, primarily crafted from polyolefin, possess a robust chemical structure, rendering them exceptionally resistant to degradation. With the continuous use of plastic film, the residual plastic film will form a large number of plastic fragments or microplastics retained in the soil after ultraviolet light irradiation and natural weathering, which leads to a series of problems, such as soil compaction (Liu et al., 2014), water and nutrient transportation obstacles (Gao et al., 2023), microbial activity reduction (Muñoz et al., 2017), agricultural operation obstruction (Gao et al., 2022), crop emergence rate reduction (Wang et al., 2023), nutrient absorption and root growth and development restriction (Chen et al., 2022), and even a reduction in yields (Li et al., 2022e), which are no longer in line with the current development direction of green and sustainable agriculture.

Studies have shown that the high air tightness of plastic film inhibits gas exchange at the soil–gas interface and leads to poor soil aeration, creating an environment where the partial pressure of soil gas carbon dioxide is too high and oxygen is lacking, which inhibits root respiration and leads to root growth restriction, yellow leaves, senescence, and even death of the plant (Zhou et al., 2024). For example, in Northwest China, poor soil aeration under drip irrigation stimulated the growth of maize roots, resulting in a shallow distribution of root growth, limiting root uptake of soil water and fertilizer, and increasing the risk of premature crop aging (Zhou et al., 2020). Plastic film mulching in the late growth stage of wheat causes membrane lipid oxidation in flag leaf cells, disrupting the balance of reactive oxygen species metabolism and the photosynthetic structure of leaves, which is not conducive to the accumulation and transportation of photosynthetic products (Zhang et al., 2023b). These phenomena are due to the generally thick plastic film thickness, resulting in poor air permeability, which is why China mandated the use of 0.01-mm ultra-thin polyethylene plastic film in 2020. Although weeds are more likely to break through the plastic film in the middle and late stages of crop growth, thus improving the air permeability, there is still a phenomenon of crop root rot (Guo et al., 2023a).

Therefore, researchers have focused on other environmentally friendly materials to replace plastic film, through the rational use of environmentally friendly materials not only to play a role in water conservation and moisture-maintaining but also to avoid the problem of environmental pollution, in which oxo-biodegradable plastic film, wheat straw, and other biological resources and mineral resources such as aubergine-super absorbent polymers (A-SAP) all retaining water and stabilizing the temperature and fertilization of the soil have been popularized and applied in agricultural production in some areas. Although, in many crop productions, it has been demonstrated that covering oxo-biodegradable plastic film and wheat straw or applying A-SAP reduces inter-plant evaporation, increases soil water storage, improves soil temperatures, improves the quality of arable land, and promotes crop growth, thereby increasing precipitation use efficiency and crop yield. Among them, oxo-biodegradable plastic film is favored by agricultural producers because it has the same heat and moisture-maintaining effect and the same mechanical properties as ordinary plastic film, and it can be completely degraded and will not pollute the soil. China, as the largest agricultural country in the world in terms of production and use of plastic agricultural film, is concerned about whether oxo-biodegradable plastic film can replace ordinary plastic film.

With high nutritional value and economic value, purple garlic has been cultivated in a large area in the China Hexi Corridor oasis agricultural area for a hundred years, forming a large-scale, scientific, and industrialized development, and has become a pillar industry for farmers to increase income and rural economic development. Among them, ground mulching, as an important cultivation measure, plays an important role in increasing soil physicochemical properties and improving the yield and quality of garlic and has been widely used in garlic cultivation. Ground-mulching materials mainly include plastic film, straw, and decomposed sheep manure (Li et al., 2021b). Yu and Yang (1988) have confirmed that the use of polyethylene plastic film for ground mulching can significantly increase the yield of garlic. However, due to the late growth of garlic, the long-term high-temperature and high-humidity environment under the plastic film is not conducive to bulb growth and quality accumulation and even large areas of “aerial garlic,” resulting in yield reduction. Li (2023) showed that the use of decomposed sheep manure to mulch garlic farmland could obtain the same yield as that of plastic film mulching and showed good ecological benefits. Hu et al. (2023) showed that the use of biodegradable plastic film mulching to obtain high yield could also improve the soil structure of garlic farmland, increase the soil active organic carbon components and the organic carbon content of >0.25-mm water-stable aggregates, and contribute to the improvement of soil quality. Li et al. (2021b), by comparing the effects of white plastic film, black plastic film, straw mulching on the quality of farmland soil, and the yield and quality of garlic, found that the mulching measures could all improve the soil enzyme activity, increase the yield of garlic, and improve the quality of bulbs, among which the comprehensive effect of white plastic film mulching was superior than that of other mulching measures. Therefore, moisture-maintaining measures should be rationally selected to ensure garlic production and quality safety while improving soil quality and to realize the sustainable development of agroecosystem. Because different soil moisture-maintaining measures have great differences on soil moisture, temperature, nutrients, microbial quantity, enzyme activity, and other physicochemical properties, different moisture-maintaining measures make significant changes in the soil micro-region environment and affect garlic growth and dry matter accumulation, especially in the cold and drought conditions. What changes do different soil moisture-maintaining measures have on soil physicochemical properties and nutrient status of garlic? Which moisture-maintaining measures are more suitable for garlic production in the cool irrigation area? There is still a lack of in-depth research to truly achieve water conservation and moisture maintaining, soil improvement, and stable yield and quality improvement. Focusing on the above problems, under different moisture-maintaining measures, this paper researched the soil water and heat condition of garlic in different growth stages and the soil micro-region environmental changes after each harvest; analyzed the interrelationships among soil moisture, temperature, nutrients, and microbial and enzyme activities through comparative experiments; and evaluated the soil quality on the basis of the TOPSIS method. Combined with the principal component analysis, the effects of moisture-maintaining measures on garlic yield, quality, and water–fertilizer productivity were comprehensively evaluated, and the best moisture-maintaining measures for garlic in the cool irrigation area were defined, which provided theoretical basis and application reference for the sustainable development of garlic industry in the China Hexi Corridor oasis agricultural area.

## 2 Materials and methods

### 2.1 Experimental site profile

**Figure 1:**
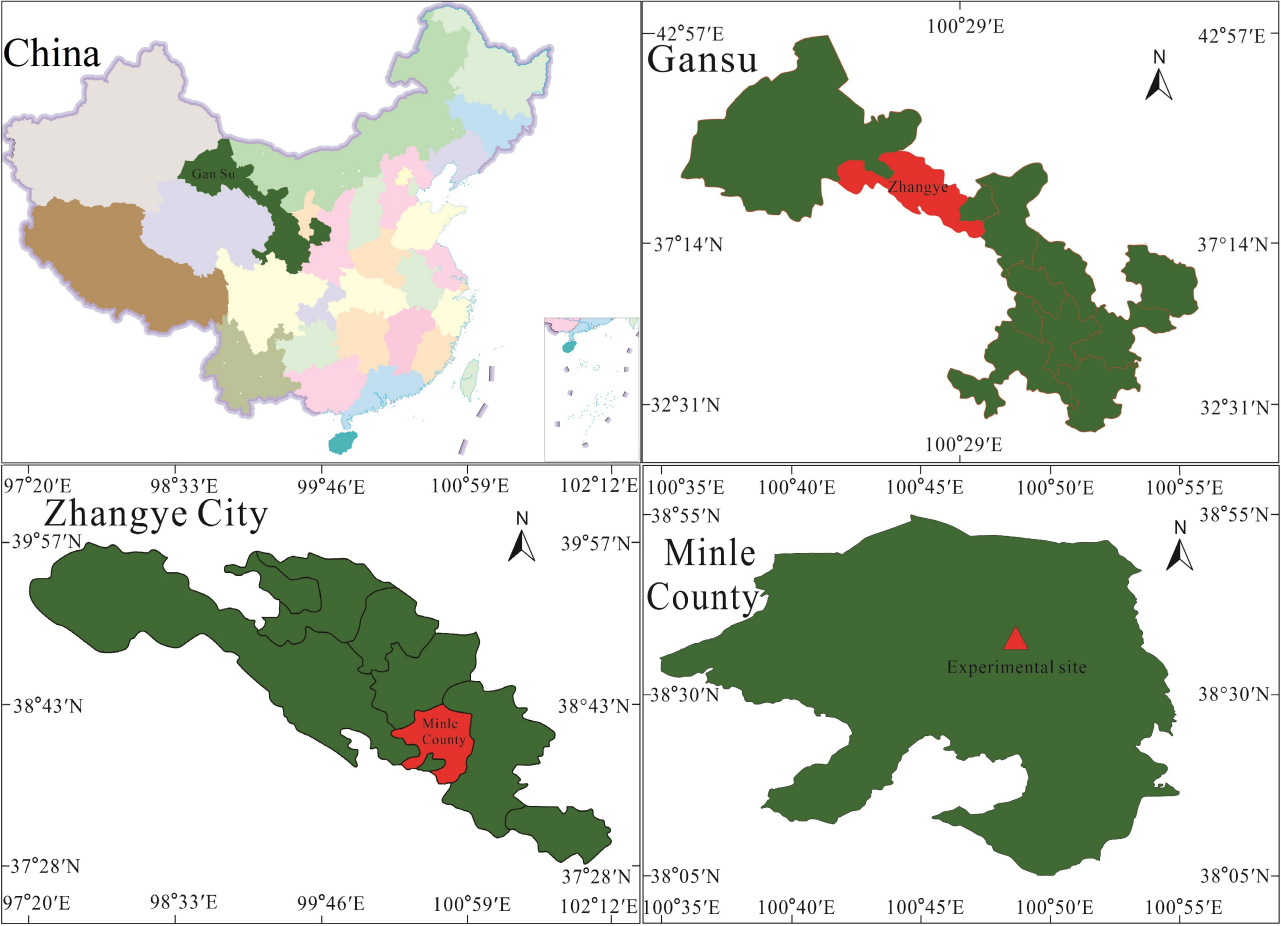
Location of the experimental site.

**Figure 2:**
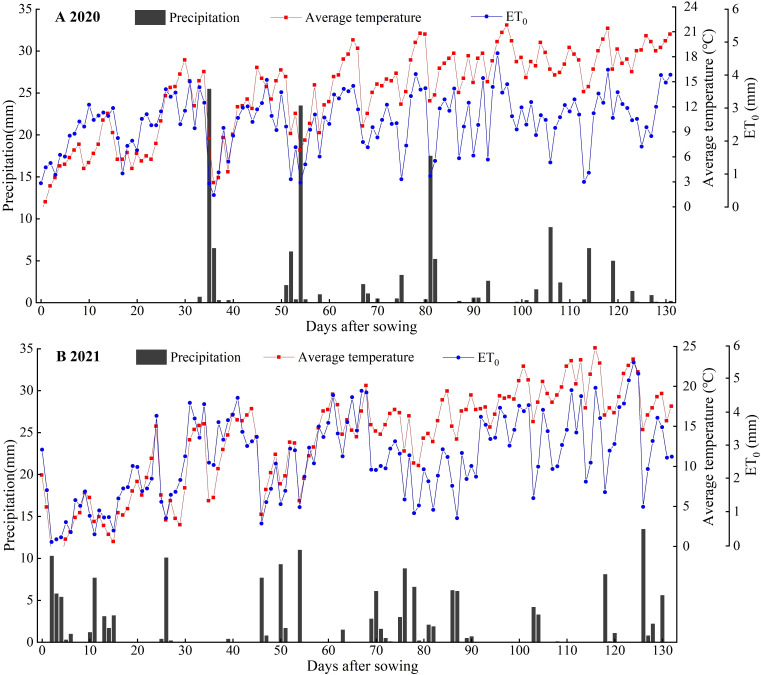
Daily variation of average temperature, reference crop evapotranspiration (ET_0_) and precipitation throughout the purple garlic growing seasons of 2020 **(A)** and 2021 **(B)**.

The experimental site is located in Yimin irrigation experimental station, Minle county, the middle part of the Hexi Corridor, Gansu province (100°43′E, 38°39′N) (**Figure 1**). The experimental area belongs to the edge area of the Qinghai-Tibetan Plateau, in the water conservation area at the Northern of the Qilian Mountains. It has an altitude of 1,970 m, an annual average temperature of 7.6°C, an effective cumulative temperature of ≥10°C of 2,985°C, an annual average of 2,932 h of sunshine, a frost-free stage of about 109~174 days, an average annual precipitation of about 200 mm, and an evaporation of 1,638 mm, which belongs to the typical continental desert-steppe climate. The field water capacity (θ_f_) is 24.0%, and the region has a deeper buried water table and less salinization impact and is characterized by less and unevenly distributed rainfall and shortage of river source, leading to outstanding supply–demand contradictions and frequent drought. The effective precipitation during the growth stage of purple garlic in 2020 was 67.88 mm and in 2021 was 111.91 mm. The basic physicochemical properties of the soil in the experimental site before planting are shown in **Table 1**, and the precipitation and average temperature during the experiment are shown in **Figure 2**.

**Table 1:** Basic physicochemical properties of 0-40 cm soil layer in the experimental site.

Soil type	Soil depth(cm)	OM (g kg^−1^)	TN (g kg^−1^)	TP (g kg^−1^)	TK (g kg^−1^)	NN (mg kg^−1^)	AN (mg kg^−1^)	AP (mg kg^−1^)	AK (mg kg^−1^)	pH	BD (g cm^−3^)
Light loam	0–20	14.5	1.23	1.11	23.5	190.50	24.60	113.7	751	8.16	1.43
	20–40	13.8	1.14	0.94	22.5	89.15	23.20	56.2	669	8.36	1.48

OM, soil organic matter content; TN, soil total nitrogen content; TP, soil total phosphorus content; TK, soil total potassium content; NN, soil nitrate nitrogen content; AN, soil ammonium nitrogen content; AP, soil available phosphorus content; AK, soil available potassium content; BD, soil bulk density.

### 2.2 Experimental materials and design

#### 2.2.1 Experimental materials

Garlic: The variety was “Minle purple Garlic,” which is a high-quality garlic variety with the clove large and fat, juicy and flavorful, mellow and pungency, and storable and is supplied by Minle County Garlic Distribution Company Limited, with a planting density of 4.4 × 10^5^ plants hm^−2^.

A-SAPs: It is selected agroforestry long-term SAP (validity stage of 3 years), provided by Gansu Hairida Ecological Environment Technology Co., Ltd. It is made of attapulgite and polyacrylamide through special physical and chemical processes, which has large water-absorbing multiplier and good soil improvement effect. By improving the properties of the crop soil such as soil compaction and other problems, it can better improve the efficiency of the use of water and fertilizers in agricultural production, reduce the loss of soil nutrients, and increase the economic benefits of agricultural production. A-SAP mixed with seed fertilizer to apply into the soil before planting, with an application rate of 45 kg hm^−2^, and the application depth of 10 cm.

Polyethylene plastic film: Provided by Lanzhou Jintudi Plastic Products Co., Ltd., with polyethylene as the main raw material, it has good functions of heat preservation, moisture preservation, and insect and disease prevention, which can promote crop growth, improve yield and quality, and facilitate the recycling of crops after harvest. The plastic film width is 120 cm, the thickness is 0.02 mm, the mulching ratio is 100%, and the mulching amount is 150 kg hm^−2^.

Oxo-biodegradable plastic film: Provided by Shandong Tianzhuang Environmental Protection Technology Co., Ltd., with ordinary polyethylene base material co-blend and oxo-biodegradable additives as the main raw materials, it has good tensile, light transmission, and other use performance and also can be controlled degradation according to the growth needs of different crops. The oxidative degradation of large–molecular weight polyethylene into small–molecular weight oligomers through the action of light, heat, and microorganisms in nature, and further degradation by microorganisms in the soil into carbon dioxide, water, and humus finally returns to the ecosystem. The plastic film width is 120 cm, the thickness is 0.008 mm, the mulching ratio is 100%, and the mulching amount is 86.0 kg hm^−2^.

Wheat straw: The milled wheat straw was cut into sections of 3–5 cm in length, mulching ratio with 100%, and mulching amount with 6,000 kg hm^−2^.

Drip irrigation pipe: Provided by Dayu Irrigation Group Co., Ltd., with an inlaid patch type, bearing pressure of 0.1 Mpa, diameter of 16 mm, wall thickness of 0.3 mm, drippers spacing of 150 mm, and rated flow rate of 2.0 L h^−1^.

#### 2.2.2 Experimental design

**Figure 3:**
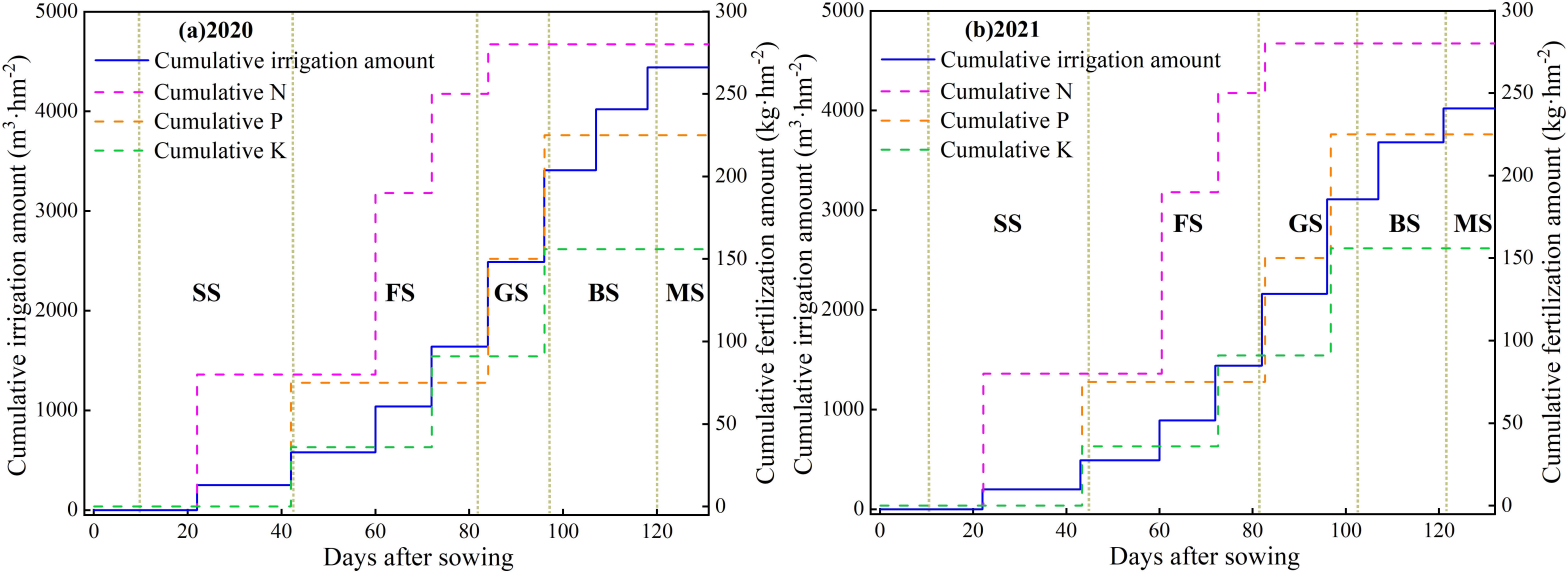
Design of different irrigation and fertilization systems of purple garlic in 2020 and 2021. SS, seedling stage; FS, flower bud differentiation stage; GS, garlic stem elongation stage; BS, bulb enlargement stage; MS, mature stage.

**Figure 4:**
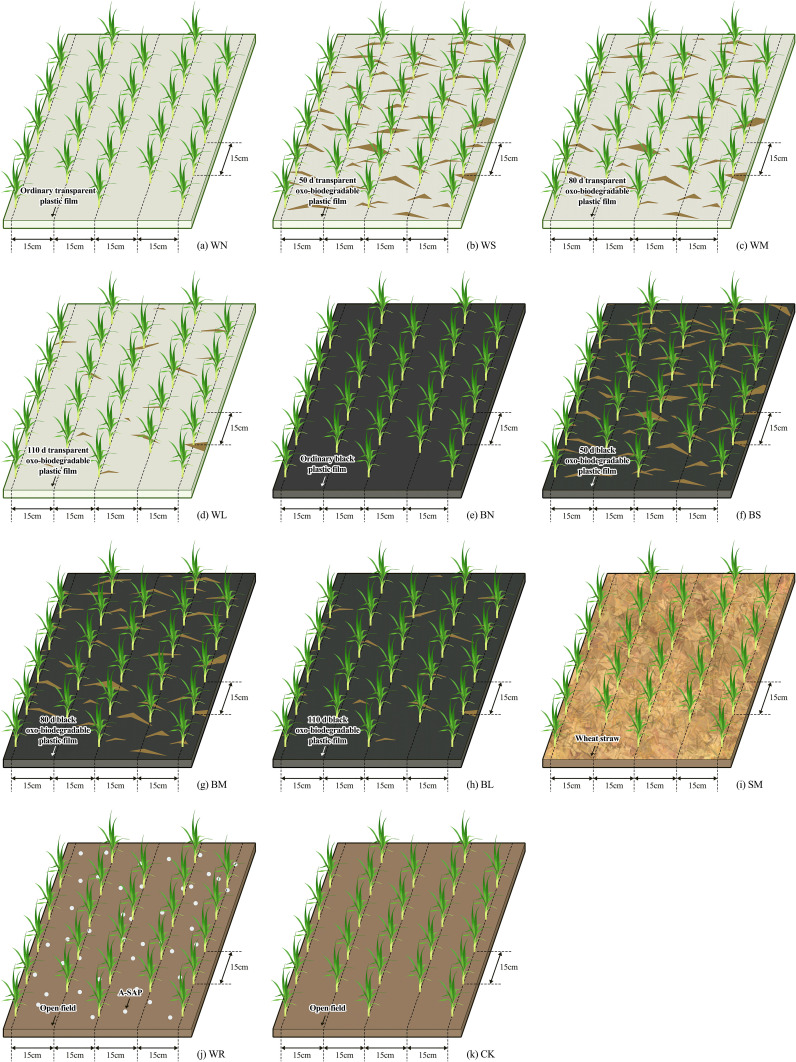
Schematic diagram of the planting mode. WN, ordinary transparent plastic film mulching; WS, 50-day transparent oxo-biodegradable plastic film mulching; WM, 80-day transparent oxo-biodegradable plastic film mulching; WL, 110-day transparent oxo-biodegradable plastic film mulching; BN, ordinary black plastic film mulching; BS, 50-day black oxo-biodegradable plastic film mulching; BM, 80-day black oxo-biodegradable plastic film mulching; BL, 110-day black oxo-biodegradable plastic film mulching; SM, straw mulching; WR, applying A-SAP; CK, no mulching and no A-SAP. The open filed is the farmland has no mulch and no A-SAP and remained in its original state.

**Figure 5:**
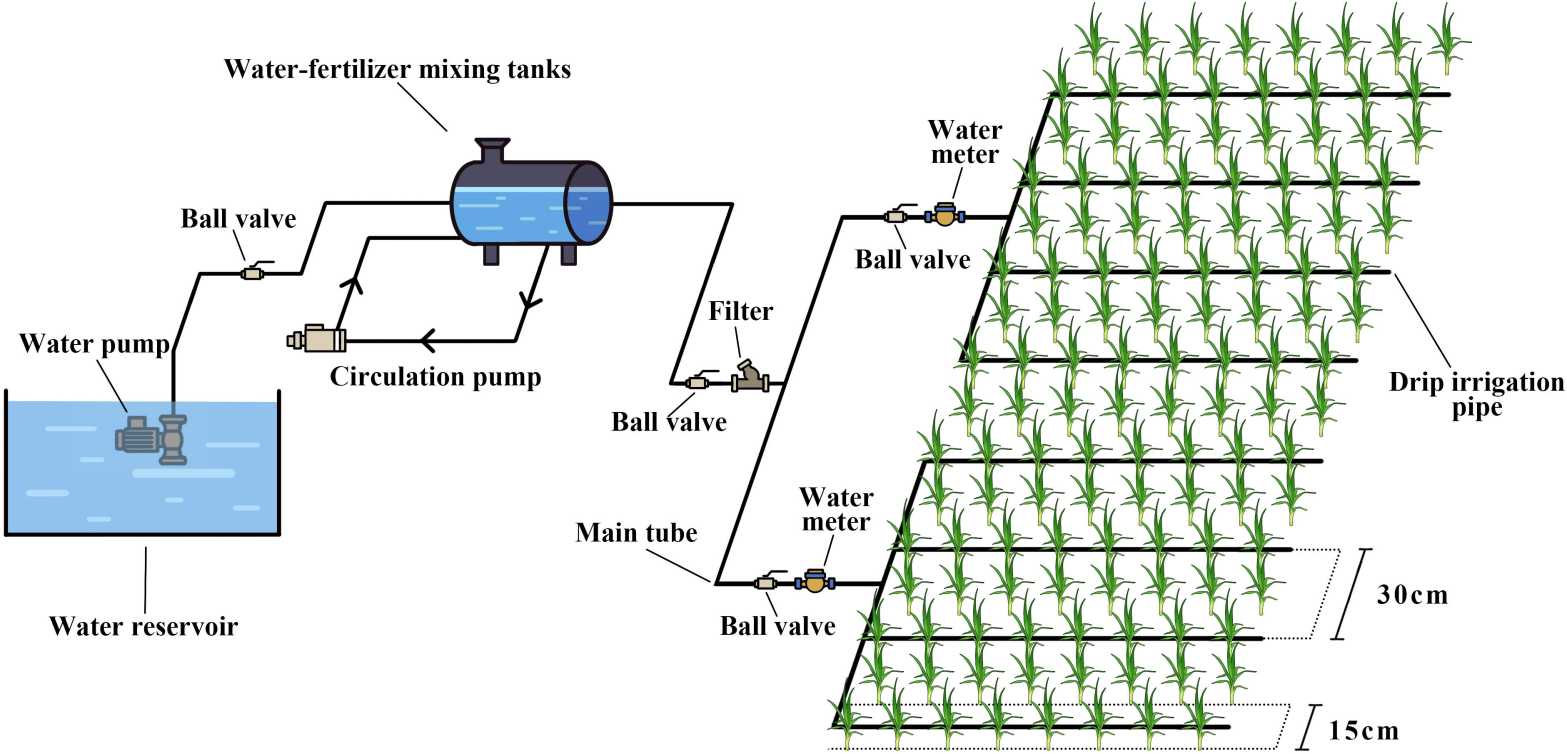
The head unit fitting drawing in the experiment.

The experiment was conducted from March to August in 2020 and 2021 with open-field no mulching and no A-SAP as control (CK), ordinary transparent plastic film mulching (WN), 50-day transparent oxo-biodegradable plastic film mulching (WS), 80-day transparent oxo-biodegradable plastic film mulching (WM), 110-day transparent oxo-biodegradable plastic film mulching (WL), 50-day black oxo-biodegradable plastic film mulching (BS), 80-day black oxo-biodegradable plastic film mulching (BM), 110-day black oxo-biodegradable plastic film mulching (BL), straw mulching (SM), and applying A-SAP (WR), a total of 11 treatments (**Figure 3**). Randomized block design was adopted in the experiment, each treatment was repeated three times, with a total of 33 plots, and the plot area was 5.0 *m* × 10 *m* = 50 m^2^. Surface-water (electrical conductivity, 5.1 μS cm^−1^; total salt content, 376 mg L^−1^; pH, 7.9) was used as the irrigation source. Each plot was installed with an independent ball valves, filters, and water meter to control the amount of irrigation; pressure differential fertilization tanks were used for fertilization. Filters were 120-mesh, and accuracy of water meters was 0.0001 m^3^ (**Figure 4**). Urea (N ≥ 46%; 610 *kg* hm^−2^ *)*, diammonium phosphate (P_2_O_5_ ≥ 42%; 536 *kg* hm^−2^), and potassium sulfate (K_2_O ≥ 52%; 300 *kg* hm^−2^) were used for seed fertilizer. All treatments were irrigated eight times throughout the growth period, and the dates and times of irrigation and fertilized are shown in **Figure 5**. Garlic was cropped for 2 years in a row, and the experimental plots were stable. The 2020 experimental was sown on 1 April and harvested on 10 August. The 2021 experimental was sown on 29 March and harvested on 8 August.

### 2.3 Measurement items and methods

#### 2.3.1 Soil water storage

Soil samples of 0–20, 20–40, 40–60, 60–80, and 80–100 cm were taken during the garlic seedling stage, scaly bud and flower bud differentiation stage, garlic bolt elongation stage, bulb swelling stage, and mature stage, respectively, and the soil water content was determined by traditional drying and weighing method, and, then, the soil water storage was calculated.

The formula for calculating soil water storage is: M = h × ρ × ω × 10, where M is the soil water storage (mm), h is the soil layer depth (cm), ρ is the soil bulk density (g cm^−3^), and ω is the soil water content (%).

#### 2.3.2 Soil temperature

Soil temperatures of 5-, 10-, 15-, 20-, and 25-cm soil layers were measured by right-angle mercury geothermometer (−30°C~50°C, accuracy of 1°C) at the garlic seedling stage, scaly bud and flower bud differentiation stage, garlic bolt elongation stage, bulb swelling stage, and mature stage, respectively. In order to ensure the representativeness of the data, one set was arranged in the front, middle, and rear positions of each plot, and the observations were made every 2 h from 8:00 am to 20:00 pm every day. Each growth stage was observed continuously for 5 days, and the average value was taken.

#### 2.3.3 Soil nutrients

Soil samples of 0- to 20-cm and 20- to 40-cm soil layers were taken after garlic harvesting according to the five-point method, and the contents of soil organic matter, total nitrogen, total phosphorus, total potassium, nitrate nitrogen, ammonium nitrogen, available phosphorus, and slow-release potassium were determined after air drying. Soil organic matter was determined by external heating with potassium dichromate-concentrated sulfuric acid, total nitrogen by semimicro-Kjeldahl determination, total phosphorus by molybdenum antimony colorimetric method, total potassium by flame photometric method, nitrate nitrogen by ultraviolet spectrophotometry method, ammonium nitrogen by indophenol blue colorimetric method, available phosphorus by molybdenum antimony colorimetric method, and slow-release potassium by ammonium acetate extraction-flame photometric method (Lu, 2000).

#### 2.3.4 Soil microbial measurement

Soil samples of 0- to 20-cm and 20- to 40-cm soil layers were taken after garlic harvesting according to the five-point method, and soil microbial quantity was determined immediately. Bacterium were determined by beef extract peptone agar medium method, fungi by Martin-Bengal red agar medium method, and actinomycetes by modified Gau No. 1 agar medium method (Wang et al., 2017a). Soil microbial biomass carbon and soil microbial biomass nitrogen were determined by chloroform fumigation–K_2_SO_4_ extraction method (Wu et al., 2006).

#### 2.3.5 Soil enzyme activity measurement

Soil samples of 0- to 20-cm and 20- to 40-cm soil layers were taken after garlic harvesting according to the five-point method, and the soil enzyme activity was determined after air-drying. Urease activity was determined by the sodium phenol-sodium hypochlorite colorimetric method, sucrase activity by the 3,5-dinitrosalicylic acid method, catalase activity by the KMnO_4_ titrimetric method, and alkaline phosphatase activity by the disodium benzene phosphate colorimetric method (Guan, 1986).

#### 2.3.6 Yield and quality measurement

After the garlic was mature, it was individually harvested by plot and then transported to a sunshade for drying, and left to dry for 2–3 days to remove the pseudostems, retain the bulbs and calculate the yield, and plot yields were converted to kg hm^−2^. At the same time, 10 garlic plants were randomly taken from each plot for quality determination of allicin, soluble sugar, soluble protein, vitamin C, crude fiber, ash content, and amino acid. Allicin was determined by Ultra Performance Liquid Chromatography (UPLC) method (Wang et al., 2024), soluble sugar by anthrone colorimetric method (Wang et al., 2024), soluble protein by Kaomas Brilliant Blue G-250 method (Wang et al., 2024), vitamin C by red phenanthroline colorimetric method (Wang et al., 2024), crude fiber by filter-bag method (Zhou et al., 2023), and ash content by reference to GB-5009.4-2016 (Guo et al., 2023b), and amino acid was performed in accordance with the operating procedures of the kit manual (Zhou et al., 2023).

#### 2.3.7 Water productivity and irrigation water productivity

Water productivity is calculated as WP = Y·ET^−1^·10^−1^, where WP is water productivity (kg m^−3^), Y is garlic yield (kg hm^−2^), ET is the garlic water consumption during the whole growth stage, and ET (mm) = P + I + B − A, where P is the rainfall during the growth stage, I is the irrigation amount during the growth stage, and B and A are the 0- to 100-cm soil water storage before planting and after harvesting, respectively.

Irrigation water productivity is calculated as IP = Y·I^−1^·10^−1^, where IP is irrigation water productivity (kg m^−3^), Y is garlic yield (kg hm^−2^), and I is the irrigation amount during the growth stage (mm).

#### 2.3.8 Nitrogen, phosphorus, and potassium fertilizer partial factor productivity

 Nitrogen fertilizer partial factor productivity (NP, kg kg**^−^** ^1^) = garlic yield/nitrogen application amount.

 Phosphorus fertilizer partial factor productivity (PP, kg kg**^−^** ^1^) = garlic yield/phosphorus application amount.

 Potassium fertilizer partial factor productivity (KP, kg kg**^−^** ^1^) = garlic yield/potassium application amount.

### 2.4 Data statistics and analysis

Microsoft Excel 2019 (Microsoft Corp., Raymond, Washington, DC, USA) software was used to for initial data checking and calculations. SPSS Statistics 24.0 (IBM, Inc., New York, NY, USA) was used to analyze the variability in data for each treatment, and Origin Pro 8.0 (Origin Lab, Corp., Hampton, MA, USA) software was used for plotting. Yaaph v12.5.7528.33196 (Meta Decision Software Technology Co., Ltd., Corp., Shanxi, China) software was used to draw the comprehensive analytical hierarchical model of the weight analysis of each index; Matlab (Version R2023b, MathWorks, Corp., Natick, MA, USA) was used to calculate the weights of soil moisture conservation measures based on game theory and the comprehensive score of TOPSIS.

## 3 Results and analysis

### 3.1 Effects of different moisture-maintaining measures on the soil environment

#### 3.1.1 Effects of different moisture-maintaining measures on the soil water storage during garlic growth stage

**Figure 6:**
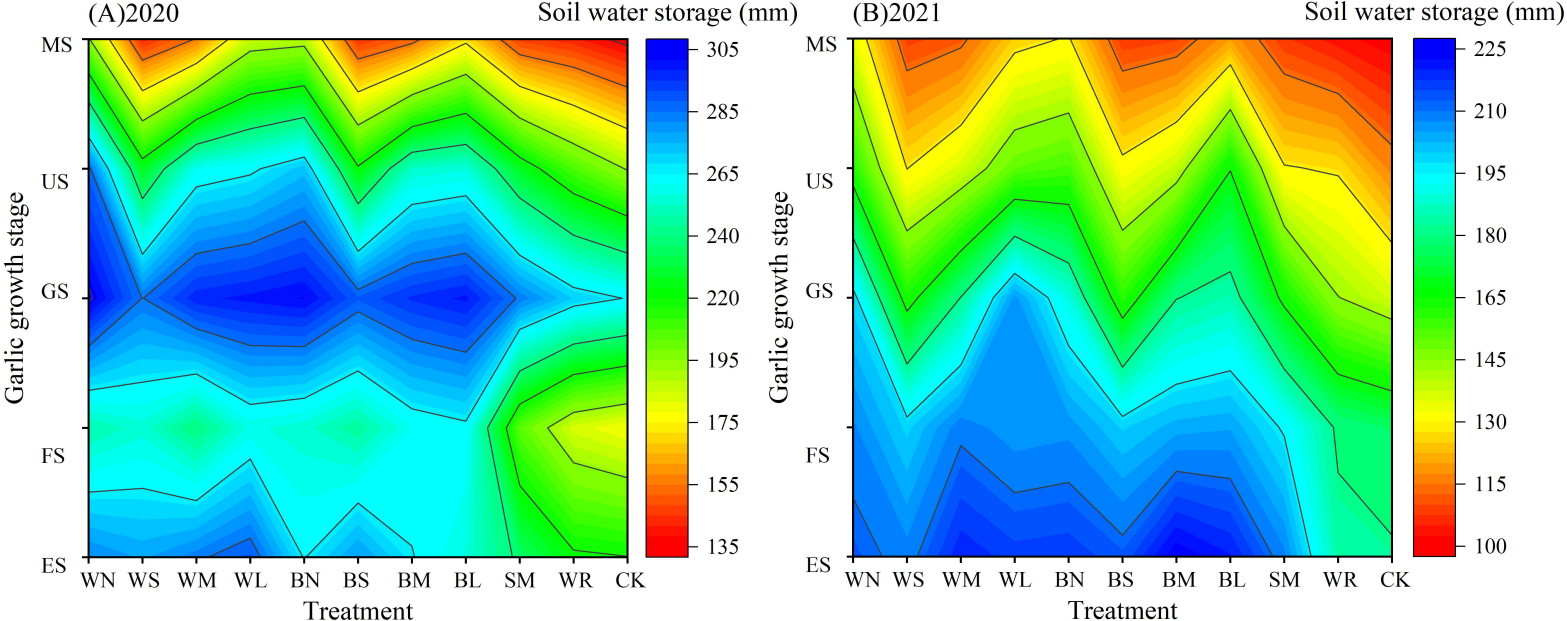
Dynamic changes of soil water storage in different growth stages of garlic under different soil moisture-maintaining measures.

Soil water storage is not only closely related to irrigation amount and precipitation but also affected by the water consumption of garlic growth and continuation. The experimental years showed the same trend of change, both increasing with irrigation or precipitation replenishment and showing a decreasing trend with garlic growth and continuation (**Figure 6**). In a comprehensive analysis of 2-year experiment result, all soil moisture-maintaining measures showed inhibiting evaporation effects, effectively increasing soil water storage by 0.77% to 48.59% compared with that of CK. However, the soil moisture-maintaining effects of each measure varied at different growth stages of garlic. There was no significant difference between soil water storage in plastic film mulching at seedling stage and scaly bud and flower bud differentiation stage, which increased significantly by 14.92% to 31.57% and 12.98% to 46.86%, respectively, compared with that of CK. During the bulb swelling stage, which was in the high temperature with intense evaporation, the WS and BS had already entered the dehiscence period, resulting in a significant reduction of 21.13% and 18.63% in soil water storage compared with that of WN but still a significant increase of 16.69% and 14.15% compared with that of CK. At this time, SM showed better moisture-maintaining effect than WR, with a significant increase of 17.03% in soil water storage compared with that of CK. At mature stage, the WS and BS had entered the macrofracture period; the WM and BM entered the dehiscence period; and the soil water storage was significantly reduced by 28.09%, 25.02%, 20.69%, and 20.44% compared with that of WN, whereas the WM and BM were still significantly higher by 17.85% and 14.85% compared with that of the CK. During this stage, the WN and BN had the best moisture-maintaining effect and the highest soil water storage, followed by the WL and BL, whereas the SM and WR did not show a significant moisture increase effect. It can be seen that the use of moisture-maintaining measures was all effective in maintaining moisture, with plastic film mulching being the most effective in maintaining moisture, followed by SM, and the WR increases the least. In the oxo-biodegradable plastic film–mulching treatments, the moisture-maintaining effect was gradually weakened as the oxo-biodegradable plastic film entered the induction period, dehiscence and macrofracture period.

#### 3.1.2 Effects of different moisture-maintaining measures on the soil temperature during garlic growth stage

**Figure 7:**
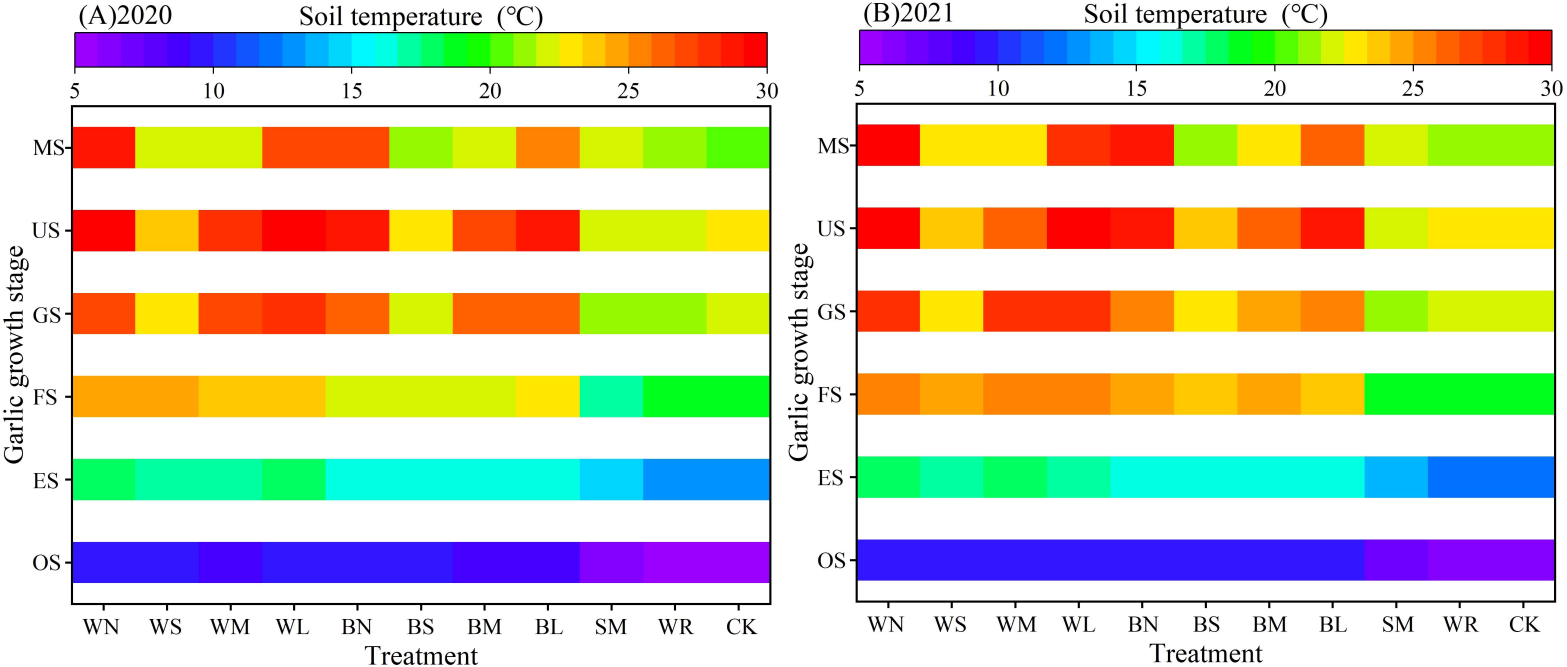
Dynamic changes of soil temperature in different growth stages of garlic under different soil moisture-maintaining measures.

The effects of moisture-maintaining measures on soil temperature showed the same trend in the two experimental years, both increasing with the increase of atmospheric temperature and continuous decreasing with the increase of pyrolysis degree under the condition of oxo-biodegradable plastic film mulching (**Figure 7**). During the garlic sowing and seedling stages, all the mulching measures significantly increased the average soil temperature in the 0- to 25-cm soil layer compared with CK, with increases of 14.70% to 69.44% and 10.28% to 44.15%, respectively. In addition, there was no significant difference among all the plastic film–mulching treatments during the sowing stage, but it was significantly higher than that of the SM. During the scaly bud and flower bud differentiation stage, the heat-maintaining effect of plastic film–mulching treatments was significant, which was significantly increased by 18.07% to 33.11% compared with that of CK. During the garlic bolt elongation stage, WS and BS entered the mid-induction period, which was significantly decreased compared with other plastic film–mulching treatments. During the bulb swelling stage, WM and BM entered the initial-induction period, which were still significantly increased by 14.77% to 22.42% and 13.52% to 19.52% compared with that of CK. However, the WS and BS had entered the dehiscence period, which was significantly lower than that of other plastic film–mulching treatments. During the mature stage, WS and BS had entered the macrofracture period, WM and BM entered the dehiscence period, showing no warming effect, whereas WL and BL were at the early stage of induction, which was significantly higher than that of CK, by 29.09% to 29.80% and 19.73% to 23.45%. SM did not show warming effect from scaly bud and flower bud differentiation stage to mature stage, whereas WR and CK had no significant difference in soil temperature during the whole growth stage. It can be seen that the plastic film has the best warming effect, WN is better than BN, followed by SM, whereas WR had no significant effect on soil temperature.

#### 3.1.3 Effects of different moisture-maintaining measures on the soil nutrients during garlic growth stage

Soil nutrient content varied as a function of moisture-maintaining measures, with similar trends in the effects of moisture-maintaining measures on soil nutrient content in the two experimental years (**Table 2**). Compared with CK, in 0- to 20-cm soil layer, organic matter, total nitrogen, total phosphorus, total potassium, nitrate nitrogen, ammonium nitrogen, available phosphorus, and slow-release potassium contents were significantly increased by using soil moisture-maintaining measures, by up to 10.83% to 59.17%, 11.26% to 80.13% and 22.56% to 77.44%, 18.18% to 33.81%, 22.99% to 72.12%, 58.05% to 192.92% and 50.63% to 233.44%, and 12.21% to 28.14%, respectively. In the 20- to 40-cm soil layer, organic matter, total nitrogen, total phosphorus, total potassium, nitrate nitrogen, and slow-release potassium contents were increased by using soil moisture-maintaining measures, by up to 10.66% to 34.43%, 6.08% to 30.41%, 7.86% to 45.71%, 22.09% to 55.21%, 19.75% to 105.49%, and 16.01% to 22.71%, respectively. Between the same colored biodegradable plastic films, organic matter, total phosphorus, nitrate nitrogen, and slow-release potassium accumulation gradually increased, whereas total nitrogen and ammonium nitrogen accumulation gradually decreased with the prolongation of the induction period.

**Table 2:** Effect of different moisture-maintaining measures on soil nutrients in the 0-40 cm soil layer of purple garlic farmland.

Year	Soil depth (cm)	Treatment	Organic matter (g kg^−1^)	Total nitrogen (g kg^−1^)	Total phosphorus (g kg^−1^)	Total potassium (g kg^−1^)	Nitrate Nitrogen (mg kg^−1^)	Ammonium nitrogen (mg kg^−1^)	Available Phosphorus (mg kg^−1^)	Slowly available potassium (mg kg^−1^)
2020	0–20	WN	17.9 ± 0.30bc	0.86 ± 0.03c	1.05 ± 0.02c	21.5 ± 0.29e	27.76 ± 0.77b	4.80 ± 0.17g	40.4 ± 0.89b	700 ± 11.81a
		WS	14.7 ± 0.48f	0.81 ± 0.02cd	0.91 ± 0.02e	23.3 ± 0.42bc	22.13 ± 0.66d	7.45 ± 0.16d	37.8 ± 1.21b	676 ± 14.94a
		WM	15.6 ± 0.43ef	0.83 ± 0.02c	0.97 ± 0.02de	22.1 ± 0.36de	24.07 ± 0.67cd	5.93 ± 0.18e	39.0 ± 0.94b	683 ± 16.64a
		WL	17.1 ± 0.56cd	0.86 ± 0.01c	1.03 ± 0.03cd	21.9 ± 0.36e	26.44 ± 0.75bc	5.24 ± 0.15fg	39.5 ± 1.19b	689 ± 17.76a
		BN	19.5 ± 0.50a	0.97 ± 0.01b	1.15 ± 0.02a	22.9 ± 0.16cd	41.15 ± 1.09a	9.03 ± 0.35c	49.7 ± 1.46a	663 ± 18.99ab
		BS	16.4 ± 0.26de	1.06 ± 0.01a	1.01 ± 0.02cd	24.7 ± 0.10a	38.65 ± 1.16a	12.50 ± 0.24a	48.2 ± 0.93a	644 ± 22.62ab
		BM	18.0 ± 0.47bc	1.01 ± 0.02ab	1.08 ± 0.03bc	24.1 ± 0.35ab	38.93 ± 1.34a	10.78 ± 0.25b	48.5 ± 1.06a	651 ± 13.60ab
		BL	18.7 ± 0.66ab	0.98 ± 0.03b	1.13 ± 0.04ab	23.5 ± 0.47bc	40.28 ± 1.20a	9.66 ± 0.31c	49.3 ± 0.73a	660 ± 19.63ab
		SM	12.4 ± 0.27g	0.82 ± 0.03c	0.77 ± 0.01f	22.3 ± 0.25de	21.65 ± 0.19d	7.70 ± 0.21d	31.8 ± 1.09c	689 ± 21.76a
		WR	12.1 ± 0.39g	0.80 ± 0.02cd	0.68 ± 0.02g	21.5 ± 0.18e	14.70 ± 0.49e	5.65 ± 0.19ef	12.9 ± 0.29d	607 ± 26.79bc
		CK	11.8 ± 0.29g	0.76 ± 0.01d	0.63 ± 0.02g	19.8 ± 0.32f	11.39 ± 0.29f	3.27 ± 0.10h	10.6 ± 0.19d	552 ± 27.06c
	20–40	WN	16.7 ± 0.58a	0.73 ± 0.02de	0.91 ± 0.02cd	19.5 ± 0.43d	40.55 ± 1.38a	4.45 ± 0.14g	24.9 ± 0.61de	710 ± 13.18b
		WS	15.3 ± 0.17ab	0.58 ± 0.02f	0.86 ± 0.02de	21.9 ± 0.38ab	25.46 ± 0.80e	7.35 ± 0.21d	23.3 ± 0.46e	713 ± 21.21b
		WM	15.5 ± 0.51ab	0.62 ± 0.02f	0.88 ± 0.03de	21.3 ± 0.50bc	29.10 ± 0.75cd	6.16 ± 0.23ef	23.6 ± 0.36e	721 ± 19.17ab
		WL	16.1 ± 0.38ab	0.69 ± 0.02e	0.95 ± 0.03c	20.2 ± 0.53cd	37.42 ± 1.24b	5.73 ± 0.21f	24.1 ± 0.24e	730 ± 21.93ab
		BN	15.4 ± 0.41ab	0.78 ± 0.02cd	0.85 ± 0.2de	23.2 ± 0.62a	40.88 ± 1.04a	6.18 ± 0.23ef	27.0 ± 0.96	697 ± 11.44b
		BS	13.8 ± 0.45cd	0.91 ± 0.03a	0.71 ± 0.02f	22.7 ± 0.31ab	37.13 ± 0.79b	8.55 ± 0.27c	26.6 ± 0.39d	735 ± 13.71ab
		BM	14.9 ± 0.43bc	0.88 ± 0.01ab	0.74 ± 0.01f	22.9 ± 0.66ab	38.40 ± 0.34ab	7.16 ± 0.15d	25.4 ± 0.45de	748 ± 14.26ab
		BL	15.0 ± 0.36bc	0.81 ± 0.02c	0.82 ± 0.02e	23.4 ± 0.68a	40.25 ± 1.16a	6.64 ± 0.21de	25.1 ± 0.42de	749 ± 16.24ab
		SM	15.3 ± 0.45ab	0.94 ± 0.02a	1.12 ± 0.02b	22.5 ± 0.66ab	30.80 ± 0.74c	11.50 ± 0.39a	40.8 ± 0.90b	705 ± 17.34b
		WR	16.2 ± 0.48ab	0.94 ± 0.03a	1.20 ± 0.01a	22.5 ± 0.65ab	26.50 ± 0.66de	9.90 ± 0.29b	70.2 ± 1.37a	777 ± 24.80a
		CK	13.5 ± 0.35d	0.82 ± 0.02bc	0.89 ± 0.03cd	19.3 ± 0.46d	20.37 ± 0.68f	6.11 ± 0.22ef	36.5 ± 1.23c	614 ± 15.04c
2021	0–20	WN	18.9 ± 0.32a	1.41 ± 0.03bc	1.23 ± 0.05a	20.1 ± 0.56c	91.33 ± 2.37b	4.13 ± 0.11bcd	56.8 ± 2.13a	780 ± 16.11a
		WS	15.3 ± 0.40cd	1.08 ± 0.04e	1.12 ± 0.02bc	22.8 ± 0.51a	77.75 ± 1.26d	4.60 ± 0.14a	47.1 ± 1.23c	747 ± 20.84ab
		WM	16.5 ± 0.54bc	1.13 ± 0.04de	1.19 ± 0.03ab	22.1 ± 0.36ab	84.91 ± 3.06c	4.39 ± 0.05ab	52.0 ± 1.80b	759 ± 22.76a
		WL	18.2 ± 0.53a	1.37 ± 0.02c	1.25 ± 0.03a	20.9 ± 0.63bc	89.26 ± 2.02bc	4.27 ± 0.06bc	54.3 ± 2.08ab	776 ± 21.65a
		BN	18.7 ± 0.33a	1.31 ± 0.02c	1.21 ± 0.04a	20.2 ± 0.64c	49.16 ± 0.32g	3.74 ± 0.09e	57.0 ± 0.61a	675 ± 19.30c
		BS	15.3 ± 0.26cd	1.66 ± 0.06a	1.08 ± 0.02bc	22.4 ± 0.64ab	46.45 ± 0.75g	4.05 ± 0.10cd	52.7 ± 1.54ab	654 ± 17.34cd
		BM	16.9 ± 0.10b	1.51 ± 0.02b	1.11 ± 0.03bc	21.0 ± 0.57bc	47.82 ± 1.61g	3.94 ± 0.07de	53.3 ± 1.23ab	661 ± 19.80cd
		BL	19.1 ± 0.41a	1.20 ± 0.04d	1.19 ± 0.03ab	20.7 ± 0.35bc	48.03 ± 1.64g	3.91 ± 0.14de	55.8 ± 1.10ab	670 ± 23.12c
		SM	15.3 ± 0.55cd	0.86 ± 0.02f	0.99 ± 0.03c	23.5 ± 0.34a	64.10 ± 0.85e	4.15 ± 0.07bcd	43.2 ± 0.82c	654 ± 19.43cd
		WR	14.5 ± 0.23d	0.89 ± 0.03f	0.95 ± 0.01c	20.7 ± 0.76bc	101.95 ± 1.45a	4.05 ± 0.11cd	35.3 ± 0.91d	689 ± 16.20bc
		CK	12.2 ± 0.40e	0.75 ± 0.02g	0.70 ± 0.02d	15.4 ± 0.38d	57.80 ± 1.05f	2.38 ± 0.08f	21.4 ± 0.70e	603 ± 20.16d
	20–40	WN	16.1 ± 0.36ab	1.20 ± 0.02a	1.06 ± 0.03ab	20.3 ± 0.25e	60.57 ± 1.36ab	3.25 ± 0.04c	25.6 ± 0.49a	752 ± 25.27a
		WS	13.7 ± 0.41de	1.03 ± 0.03b	0.87 ± 0.02e	22.0 ± 0.16c	45.10 ± 1.27d	3.80 ± 0.10b	23.1 ± 0.67b	740 ± 14.67a
		WM	15.1 ± 0.36bc	1.04 ± 0.02b	0.95 ± 0.03cd	20.7 ± 0.39de	56.73 ± 1.91b	3.29 ± 0.04c	24.8 ± 0.67a	747 ± 23.30a
		WL	15.9 ± 0.45ab	1.18 ± 0.03a	1.09 ± 0.02a	19.6 ± 0.41e	58.29 ± 1.54b	3.13 ± 0.06cd	25.0 ± 0.55a	758 ± 23.31a
		BN	15.6 ± 0.44ab	0.79 ± 0.02de	1.00 ± 0.03bc	27.4 ± 0.57a	62.85 ± 1.26a	2.96 ± 0.07d	25.4 ± 0.72a	737 ± 14.60a
		BS	13.2 ± 0.22ef	0.83 ± 0.02d	0.80 ± 0.02f	22.5 ± 0.22c	51.10 ± 1.03c	3.60 ± 0.04b	26.3 ± 0.57a	724 ± 26.51a
		BM	15.1 ± 0.16bc	0.77 ± 0.01de	0.85 ± 0.01ef	25.3 ± 0.19b	58.43 ± 1.54b	3.08 ± 0.09cd	25.7 ± 0.47a	730 ± 27.21a
		BL	16.4 ± 0.34a	0.76 ± 0.02e	0.94 ± 0.02d	26.8 ± 0.48a	60.76 ± 0.73ab	2.74 ± 0.03e	25.0 ± 0.44a	733 ± 17.22a
		SM	14.3 ± 0.17cd	0.83 ± 0.02d	0.86 ± 0.01ef	21.5 ± 0.55cd	59.65 ± 0.99ab	4.10 ± 0.12a	21.2 ± 0.52cd	715 ± 24.63a
		WR	12.6 ± 0.33f	0.89 ± 0.02c	0.84 ± 0.03ef	19.5 ± 0.53e	33.95 ± 0.49e	3.80 ± 0.05b	22.6 ± 0.12bc	725 ± 11.59a
		CK	10.9 ± 0.21g	0.66 ± 0.01f	0.51 ± 0.01g	13.3 ± 0.18f	30.11 ± 0.90f	2.05 ± 0.06f	19.7 ± 0.17d	610 ± 19.95b
2 years average	0–20	WN	18.4 ± 0.27ab	1.14 ± 0.02c	1.14 ± 0.03abc	20.8 ± 0.36e	59.55 ± 1.54a	4.47 ± 0.12g	48.6 ± 1.41bc	740 ± 13.82a
		WS	15.0 ± 0.31c	0.95 ± 0.02d	1.02 ± 0.02e	23.1 ± 0.46ab	49.94 ± 0.96c	6.03 ± 0.15de	42.5 ± 1.20d	712 ± 17.25abc
		WM	16.1 ± 0.48c	0.98 ± 0.03d	1.08 ± 0.02cde	22.1 ± 0.34bcd	54.49 ± 1.61b	5.16 ± 0.11f	45.5 ± 1.35cd	721 ± 19.08ab
		WL	17.7 ± 0.54b	1.12 ± 0.01c	1.14 ± 0.03abc	21.4 ± 0.45cde	57.85 ± 1.36a	4.76 ± 0.11fg	46.9 ± 1.41c	733 ± 19.15a
		BN	19.1 ± 0.41a	1.14 ± 0.01c	1.18 ± 0.03a	21.6 ± 0.40cde	45.16 ± 0.62d	6.39 ± 0.22cd	53.4 ± 0.98a	669 ± 19.15bcd
		BS	15.9 ± 0.26c	1.36 ± 0.04a	1.05 ± 0.02de	23.6 ± 0.31a	42.55 ± 0.60d	8.28 ± 0.16a	50.5 ± 1.22ab	649 ± 18.10d
		BM	17.5 ± 0.30b	1.26 ± 0.02b	1.10 ± 0.02bcd	22.6 ± 0.46abc	43.38 ± 1.20d	7.32 ± 0.16b	50.9 ± 1.03ab	656 ± 13.52cd
		BL	18.9 ± 0.50a	1.09 ± 0.03c	1.16 ± 0.03ab	22.1 ± 0.39bcd	44.16 ± 1.13d	6.79 ± 0.20c	52.6 ± 0.85a	665 ± 21.36bcd
		SM	13.9 ± 0.26d	0.84 ± 0.02e	0.88 ± 0.02f	22.9 ± 0.24ab	42.88 ± 0.52d	5.93 ± 0.13e	37.5 ± 0.93e	672 ± 20.26bcd
		WR	13.3 ± 0.31d	0.85 ± 0.02e	0.82 ± 0.01f	21.1 ± 0.43de	58.30 ± 0.95a	4.85 ± 0.15fg	24.1 ± 0.59f	648 ± 13.88d
		CK	12.0 ± 0.35e	0.76 ± 0.01f	0.67 ± 0.01g	17.6 ± 0.35f	34.60 ± 0.56e	2.83 ± 0.10h	16.0 ± 0.42g	578 ± 23.54e
	20–40	WN	16.4 ± 0.46a	0.97 ± 0.01a	0.99 ± 0.02a	19.9 ± 0.34d	50.56 ± 1.37ab	3.85 ± 0.09h	25.3 ± 0.55def	731 ± 18.29a
		WS	14.5 ± 0.29de	0.81 ± 0.03ef	0.87 ± 0.02b	22.0 ± 0.26bc	35.28 ± 0.66e	5.58 ± 0.16d	23.2 ± 0.50g	727 ± 16.87a
		WM	15.3 ± 0.43abc	0.83 ± 0.02def	0.92 ± 0.03b	21.0 ± 0.44cd	42.92 ± 1.27d	4.73 ± 0.13ef	24.2 ± 0.47fg	734 ± 19.26a
		WL	16.0 ± 0.41ab	0.94 ± 0.02ab	1.02 ± 0.02a	19.9 ± 0.46d	47.86 ± 1.38bc	4.43 ± 0.13fg	24.6 ± 0.40efg	744 ± 21.91a
		BN	15.5 ± 0.38abc	0.79 ± 0.02fg	0.93 ± 0.02b	25.3 ± 0.59a	51.87 ± 1.14a	4.57 ± 0.14f	26.2 ± 0.66de	717 ± 13.01a
		BS	13.5 ± 0.28e	0.87 ± 0.02cde	0.76 ± 0.02cd	22.6 ± 0.23b	44.12 ± 0.90d	6.08 ± 0.14c	26.5 ± 0.38d	730 ± 17.77a
		BM	15.0 ± 0.27bcd	0.83 ± 0.01ef	0.80 ± 0.01c	24.1 ± 0.42a	48.42 ± 0.92b	5.12 ± 0.12e	25.6 ± 0.45def	739 ± 18.58a
		BL	15.7 ± 0.34abc	0.79 ± 0.02fg	0.88 ± 0.02b	25.1 ± 0.57a	50.51 ± 0.82ab	4.69 ± 0.12ef	25.1 ± 0.39def	741 ± 16.46a
		SM	14.8 ± 0.29cd	0.89 ± 0.02bcd	0.99 ± 0.02a	22.0 ± 0.40bc	45.23 ± 0.51cd	7.80 ± 0.26a	31.0 ± 0.70b	710 ± 20.30a
		WR	14.4 ± 0.40de	0.92 ± 0.03abc	1.02 ± 0.02a	21.0 ± 0.58cd	30.23 ± 0.49f	6.85 ± 0.17b	46.4 ± 0.67a	751 ± 18.19a
		CK	12.2 ± 0.24f	0.74 ± 0.01g	0.70 ± 0.02d	16.3 ± 0.26e	25.24 ± 0.78g	4.08 ± 0.13gh	28.1 ± 0.70c	612 ± 12.06b
ANOVA										
Year (Y)			ns	***	***	***	***	***	***	***
Treatment (T)			***	***	***	***	***	***	***	***
Soil depth (S)			***	***	***	ns	***	***	***	***
Y × T			**	***	***	***	***	***	***	ns
Y × S			***	***	***	***	***	***	***	**
T × S			***	***	***	***	***	***	***	**
Y × T × S			***	***	***	***	***	***	***	ns

Different lowercase letters in the same column indicate significant differences among different treatments (*P* < 0.05). The ** and *** indicate significant differences among different treatments at the levels of *P* < 0.01 and *P* < 0.001, respectively. The ns means not significant at the level of *P* ≥ 0.05.

#### 3.1.4 Effects of different moisture-maintaining measures on the soil microbe during garlic growth stage

**Figure 8:**
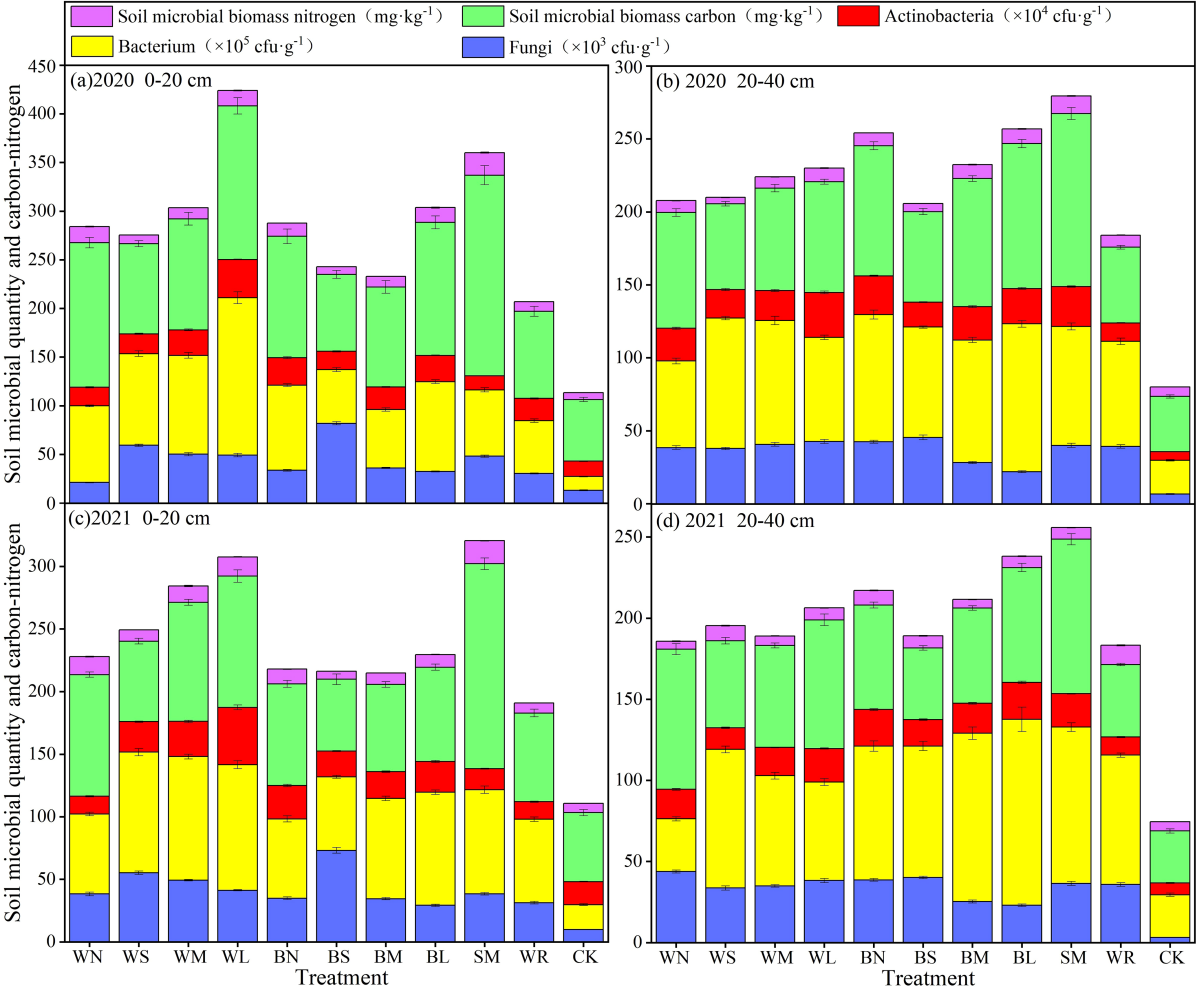
Effect of different moisture-maintaining measures on soil microbial in the 0-40 cm soil layer of purple garlic. Bars indicate standard deviations.

The effects of different moisture-maintaining measures, experimental year, and soil depth on the quantity of soil fungi, bacterium, actinobacteria, and microbial biomass carbon and nitrogen varied (**Figure 8**, **Table 3**), and, under soil moisture-maintaining measures, microbial quantity and microbial biomass carbon and nitrogen were significantly higher than that of CK. By comparing the average values of the two experimental years, it was found that all treatments could significantly increase the fungi and bacteria quantity in the 0- to 20-cm soil layer compared with that of CK, with increases of up to 159.90% to 577.63% and 233.65% to 667.88%, respectively. WN and SM inhibited soil actinobacteria reproduction, and all other moisture-maintaining measures promoted soil actinobacteria reproduction with a significant increase in quantity. Meanwhile, each treatment increased soil microbial biomass carbon and soil microbial biomass nitrogen by 15.38% to 212.80% and 0.77% to 189.00%, respectively, compared with that of CK, with the SM showing the largest increase in soil microbial biomass carbon and nitrogen. Each treatment also significantly increased the quantity of fungi, bacterium, and actinobacteria in the 20- to 40-cm soil layer compared with that of CK, with increases of up to 354.18% to 761.93%, 85.91% to 337.02%, and 81.71% to 291.12%, respectively, and the fungi quantity in BS increased by the largest. BL had the largest increase in bacterium quantity, and WL had the largest increase in actinobacteria quantity. All treatments could increase soil microbial biomass carbon and microbial biomass nitrogen, ranging from 38.12% to 205.76% and 6.89% to 64.81%, respectively, in which the increase of microbial biomass carbon was the largest in the SM, the increase of microbial biomass nitrogen was the largest in the WR.

**Table 3:** ANOVA results of different moisture-maintaining measures, experimental year, and soil depth on soil microorganism.

Factors	Fungi	Bacterium	Actinomyceteria	Soil microbial biomass carbon	Soil microbial biomass nitrogen
Year (Y)	***	*	***	***	***
Treatment (T)	***	***	***	***	***
Soil depth (S)	***	ns	***	***	***
Y × T	***	***	***	***	***
Y × S	ns	**	***	***	***
T × S	***	***	***	***	***
Y × T × S	***	***	***	***	***

Different lowercase letters in the same column indicate significant differences among different treatments (*P* < 0.05). The *, **, and *** indicate significant differences among different treatments at the levels of *P* < 0.05, *P* < 0.01, and *P* < 0.001, respectively. The ns means not significant at the level of *P* ≥ 0.05.

#### 3.1.5 Effects of different moisture-maintaining measures on the soil enzyme activity during garlic growth stage

**Figure 9:**
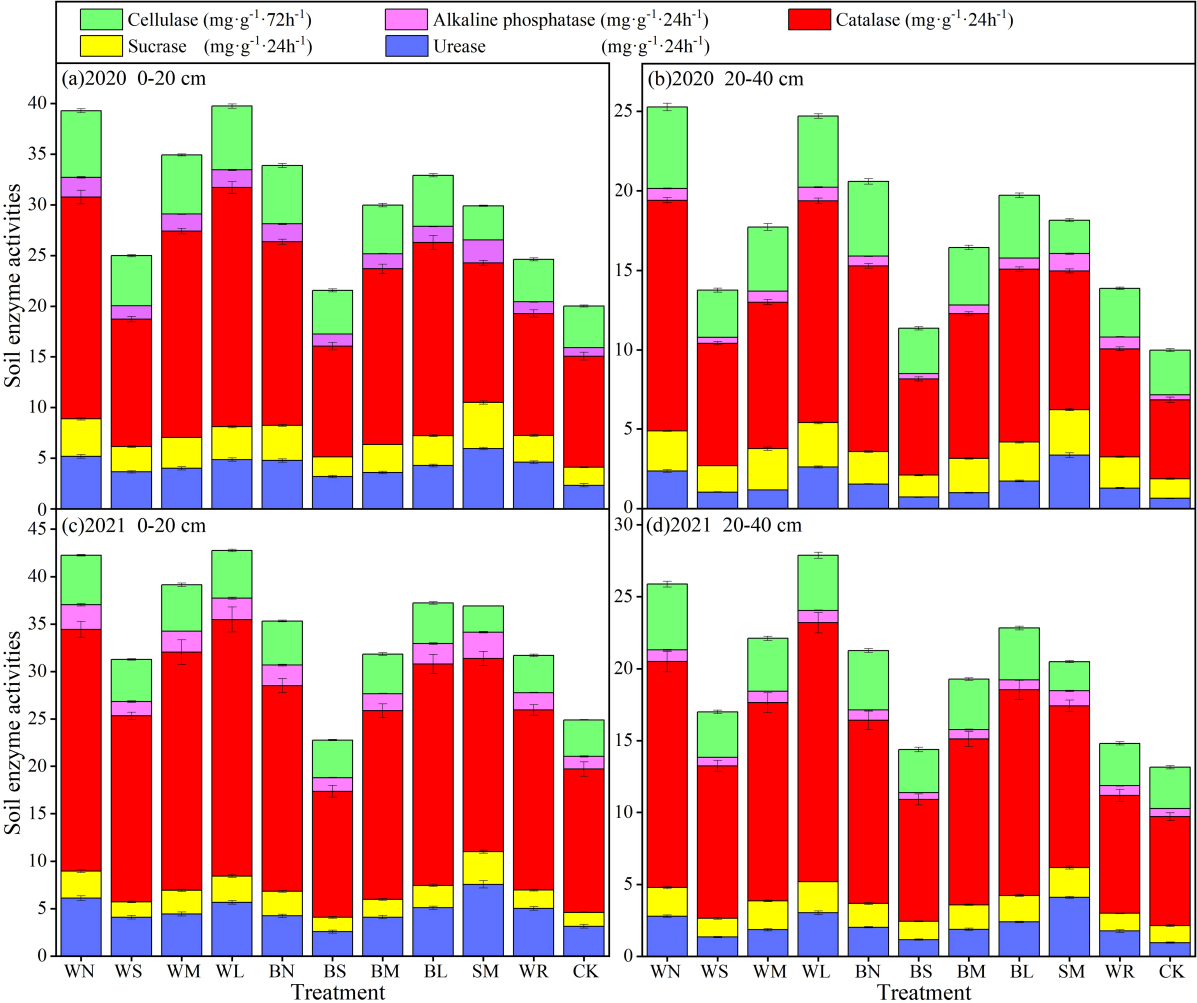
Effect of different moisture-maintaining measures on soil enzyme activities in 0-40 cm soil layer of purple garlic. Bars indicate standard deviations.

The effects of different moisture-maintaining measures, experimental year, and soil depth on the soil enzyme activities of urease, sucrase, catalase, alkaline phosphatase, and cellulase varied (**Figure 9**, **Table 4**). By comparing the average values of the two experimental years, it was found that all treatments could increase the activities of urease, sucrase, and alkaline phosphatase in the 0- to 20-cm soil layer by 5.49% to 147.07%, 6.17% to 146.91%, and 21.66% to 131.24%, respectively, compared with that of CK, among which the SM had the largest increase. There was no significant difference in soil catalase activity between BS treatment and CK treatment, and all other moisture-maintaining measures significantly increased by 19.06% to 94.21% compared with that of CK. Soil cellulase activity under SM was significantly reduced by 22.99% compared with that of CK, and other soil moisture-maintaining measures were increased by 2.01% to 48.12%, among which WN had the largest increase. Similarly, each treatment could increase the activities of soil urease, sucrase, and catalase in 20- to 40-cm soil layer, which increased by 18.24% to 368.55%, 9.96% to 104.98%, and 15.80% to 155.39%, respectively. There was no significant difference in alkaline phosphatase activity between BS treatment and CK treatment, and all other moisture-maintaining measures were significantly increased by 11.36% to 142.05%, among which SM had the largest increase. The soil cellulase activity under SM was significantly reduced by 27.80% compared with that of CK, and other soil moisture-maintaining measures were increased by 2.97% to 69.23% compared with that of CK, among which WN had the largest increase.

**Table 4:** ANOVA results of different moisture-maintaining measures, experimental year, and soil depth on soil enzyme activities.

Factors	Urease	Sucrase	Catalase	Alkaline phosphatase	Cellulase
Year (Y)	***	***	***	***	***
Treatment (T)	***	***	***	***	***
Soil depth (S)	***	***	***	***	***
Y × T	***	***	**	ns	***
Y × S	ns	***	***	***	***
T × S	***	***	***	***	***
Y × T × S	***	*	**	***	ns

Different lowercase letters in the same column indicate significant differences among different treatments (*P* < 0.05). The *, **, and *** indicate significant differences among different treatments at the levels of *P* < 0.05, *P* < 0.01, and *P* < 0.001, respectively. The ns means not significant at the level of *P* ≥ 0.05.

### 3.2 Effects of different moisture-maintaining measures on the soil quality

#### 3.2.1 Soil quality comprehensive evaluation hierarchical model

A hierarchical model for the comprehensive evaluation of soil quality was established using Yaaph software. The target layer of soil quality (C) was divided into four guideline layers: soil hydrothermal (C_1_), soil nutrients (C_2_), soil microbial (C_3_), and soil enzyme activity (C_4_); soil hydrothermal index included two index layers: water storage (C_11_) and temperature (C_12_); soil nutrient index included eight index layers: organic matter (C_21_), total nitrogen (C_22_), total phosphorus (C_23_), total potassium (C_24_), nitrate nitrogen (C_25_), ammonium nitrogen (C_26_), available phosphorus (C_27_), and slow-release potassium (C_28_); soil microbial index include five index layers: fungi (C_31_), bacterium (C_32_), actinobacteria (C_33_), microbial biomass carbon (C_34_), and microbial biomass nitrogen (C_35_); soil enzyme activity index include five index layers: sucrase (C_41_), urease (C_42_), catalase (C_43_), alkaline phosphatase (C_44_), and cellulase (C_45_).

#### 3.2.2 Index weighting

##### 3.2.2.1 AHP method

On the basis of the analytic hierarchy process (AHP) method to determine the weight hierarchical model and then the proportion of the scale method of 1 to 10 to establish the judgment matrix, and the consistency of the matrix is tested. The judgment matrix of comprehensive soil hydrothermal, soil nutrient, soil microbial, soil enzyme activity is as follows:


$$C=\left[\begin{array}{cccc} 1.0000 & 2.0000 & 3.5000 & 2.5000\\ 0.5000 & 1.0000 & 2.4000 & 3.8000\\ 0.2857 & 0.4167 & 1.0000 & 0.5000\\ 0.4000 & 0.2632 & 2.0000 & 1.0000 \end{array}\right]$$

$$C_{1}=\left[\begin{array}{cc} 1.0000 & 1.2000\\ 0.8333 & 1.0000 \end{array}\right]$$

$$C_{2}=\left[\begin{array}{cccccccc} 1.0000 & 2.0000 & 3.0000 & 3.50000 & 1.4000 & 4.5000 & 3.8000 & 4.1000\\ 0.5000 & 1.0000 & 0.5000 & 2.8000 & 1.2000 & 3.0000 & 2.5000 & 2.7000\\ 0.3333 & 2.0000 & 1.0000 & 4.0000 & 1.3000 & 4.0000 & 1.8000 & 2.1000\\ 0.2857 & 0.3571 & 0.2500 & 1.0000 & 0.3333 & 2.0000 & 0.2500 & 0.2500\\ 0.7143 & 0.8333 & 0.7692 & 3.0000 & 1.0000 & 2.5000 & 1.4000 & 1.5000\\ 0.2222 & 0.3333 & 0.2500 & 0.5000 & 0.4000 & 1.0000 & 0.5000 & 0.3333\\ 0.2632 & 0.4000 & 0.5556 & 4.0000 & 0.7143 & 2.0000 & 1.0000 & 0.5000\\ 0.2439 & 0.3704 & 0.4762 & 4.0000 & 0.6667 & 3.0000 & 2.0000 & 1.0000 \end{array}\right]$$

$$C_{3}=\left[\begin{array}{ccccc} 1.0000 & 0.3333 & 0.2500 & 0.5000 & 1.2000\\ 3.0000 & 1.0000 & 0.2500 & 2.5000 & 2.2000\\ 4.0000 & 4.0000 & 1.0000 & 3.0000 & 3.5000\\ 2.0000 & 0.4000 & 0.3333 & 1.0000 & 1.5000\\ 0.8333 & 0.4545 & 0.2857 & 0.6667 & 1.0000 \end{array}\right]$$

$$C_{4}=\left[\begin{array}{ccccc} 1.0000 & 1.5000 & 0.2500 & 0.5000 & 0.2500\\ 0.6667 & 1.0000 & 0.2000 & 0.5000 & 0.2000\\ 4.0000 & 5.0000 & 1.0000 & 2.5000 & 1.2000\\ 2.0000 & 2.0000 & 0.4000 & 1.0000 & 0.2500\\ 4.0000 & 5.0000 & 0.8333 & 4.0000 & 1.0000 \end{array}\right]$$

The consistency test coefficients C_R_ of comprehensive soil hydrothermal, soil nutrient, soil microbial, and soil enzyme activity were all less than 0.10, indicating that the consistency test results are good and that the established judgment matrix is reliable and reasonable (**Table 5**, where λ_max_ is the maximum eigenvalue). The results showed that the weights of the soil quality indices, in descending order, were water storage, temperature, organic matter, total phosphorus, cellulase, catalase, actinobacteria, total nitrogen, nitrate nitrogen, slow-release potassium, available phosphorus, bacterium, alkaline phosphatase, total potassium, microbial biomass carbon, ammonium nitrogen, sucrase, microbial biomass nitrogen, urease, and fungi.

**Table 5:** Results of AHP hierarchical analysis for calculating weights.

Hierarchy	Index	Local weight	Final weight	Consistency test parameter
Target layer	Soil hydrothermal	0.4303	0.4303	C_R_ = 0.0669 < 0.1 λmax = 4.1786
	Soil nutrient	0.3203	0.3203	
	Soil microbial	0.1036	0.1036	
	Soil enzyme activity	0.1458	0.1458	
Target layer 1	Soil water storage	0.5455	0.2347	C_R_ = 0.0000 < 0.1 λmax = 2.0000
	Soil temperature	0.4545	0.1956	
Target layer 2	Organic matter	0.2772	0.0888	C_R_ = 0.0499 < 0.1 λmax = 8.4930
	Total nitrogen	0.1493	0.0478	
	Total phosphorus	0.1701	0.0545	
	Total potassium	0.0462	0.0148	
	Nitrate nitrogen	0.1290	0.0413	
	Ammonium nitrogen	0.0407	0.0130	
	Available phosphorus	0.0850	0.0272	
	Slow-release potassium	0.1024	0.0328	
Target layer 3	Fungi	0.0878	0.0091	C_R_ = 0.0445 < 0.1 λmax = 5.1994
	Bacterium	0.2191	0.0227	
	Actinobacteria	0.4659	0.0483	
	Microbial biomass carbon	0.1333	0.0138	
	Microbial biomass nitrogen	0.0940	0.0097	
Target layer 4	Sucrase	0.0846	0.0123	C_R_ = 0.0133 < 0.1 λmax = 5.0596
	Urease	0.0655	0.0095	
	Catalase	0.3529	0.0515	
	Alkaline phosphatase	0.1312	0.0191	
	Cellulase	0.3658	0.0533	

##### 3.2.2.2 Entropy weight method

The entropy weight method was used to assign weights to a single index of soil quality, and the weights of soil quality index were calculated (**Table 6**). As can be seen from **Table 6**, the weights of the index determined by the entropy weight method are, in descending order, temperature, microbial biomass nitrogen, catalase, sucrase, urease, microbial biomass carbon, alkaline phosphatase, actinobacteria, water storage, ammonium nitrogen, cellulase, total nitrogen, organic matter, fungi, total potassium, nitrate nitrogen, bacterium, total phosphorus, slow-release potassium, and available phosphorus.

**Table 6:** Weights of single index of soil quality determined by entropy weight method.

Indices	C_11_	C_12_	C_21_	C_22_	C_23_	C_24_	C_25_	C_26_	C_27_	C_28_	C_31_	C_32_	C_33_	C_34_	C_35_	C_41_	C_42_	C_43_	C_44_	C_45_
**Weights**	0.0503	0.0801	0.0419	0.0446	0.0309	0.0319	0.0318	0.0483	0.0282	0.0285	0.0353	0.0314	0.0559	0.0648	0.0787	0.0725	0.0661	0.0747	0.0586	0.0457

##### 3.2.2.3 Game theory combinatorial empowerment

In order to improve the reliability and scientificity of the weight assignment values and to avoid the influence of subjective factors on the evaluation, a basic weight set was constructed on the basis of the two assignment values obtained by the AHP method and the entropy weight method $w=\sum_{\text{k}=1}^{l}\alpha_{k}\times w_{k}^{T}(\alpha_{k}>0)$ where *a_k_* is AHP method; *w_k_* is entropy weight method.

On the basis of the weight set model of game theory, the game model is derived $M\text{in}\left\|\sum_{j=1}^{i}a_{j}\times u_{j}^{T}-u_{i}^{T}\right\|$ *i* =1, 2. The combination coefficients after normalization of the above equation can be obtained using Matlab: a_1_ = 0.9213, a2 = 0.0787, This yields a vector of combined weights as $w^{*}=\sum_{k=1}^{2}a_{k}^{*}\times u_{k}^{T}$ , the final results are presented in **Table 7**. As can be seen from the table, the weights of the indices, in descending order, are water storage, temperature, organic matter, catalase, cellulase, total phosphorus, actinobacteria, total nitrogen, nitrate nitrogen, slow-release potassium, available phosphorus, bacterium, alkaline phosphatase, microbial biomass carbon, sucrase, total potassium, ammonium nitrogen, microbial biomass nitrogen, and urease, fungi.

**Table 7:** Soil quality single index weights determined on the basis of game theory combinatorial empowerment.

Indices	C_11_	C_12_	C_21_	C_22_	C_23_	C_24_	C_25_	C_26_	C_27_	C_28_	C_31_	C_32_	C_33_	C_34_	C_35_	C_41_	C_42_	C_43_	C_44_	C_45_
**Weights**	0.2202	0.1865	0.0851	0.0475	0.0526	0.0161	0.0406	0.0158	0.0273	0.0325	0.0112	0.0234	0.0489	0.0178	0.0151	0.0170	0.0139	0.0533	0.0222	0.0527

#### 3.2.3 Comprehensive evaluation based on the TOPSIS method

On the basis of the combination assignment TOPSIS method for comprehensive evaluation, the decision matrix was normalized, the weighting matrix was established, and the ideal solution and fit *C_i_* of the evaluation index were calculated, and the results were shown in **Table 8**. As can be seen from **Table 8**, soil quality in WL of soil moisture-maintaining measures had the largest degree of fit of the comprehensive indices (0.7798), which was optimal for comprehensive evaluation, followed by the BN and WN, whereas the WR had the lowest degree of fit, indicating that the comprehensive performance was the worst.

**Table 8:** Comprehensive indices of soil quality based on the TOPSIS method and ranking.

Treatment	C_11_	C_12_	C_21_	C_22_	C_23_	C_24_	C_25_	C_26_	C_27_	C_28_	C_31_	C_32_	C_33_	C_34_	C_35_	C_41_	C_42_	C_43_	C_44_	C_45_	D^+^	D^−^	C	Sequence
WN	0.3312	0.3439	0.3368	0.3329	0.3326	0.2815	0.3600	0.2246	0.3136	0.3168	0.2736	0.2235	0.2545	0.3464	0.3270	0.3758	0.3599	0.3884	0.3696	0.3874	0.0683	0.1233	0.6434	3
WS	0.2951	0.2982	0.2855	0.2774	0.2942	0.3112	0.2786	0.3133	0.2788	0.3097	0.3592	0.3485	0.2677	0.2267	0.2337	0.2413	0.2286	0.2507	0.2244	0.2834	0.1003	0.0808	0.4462	8
WM	0.3110	0.3179	0.3034	0.2869	0.3122	0.2980	0.3185	0.2670	0.2960	0.3134	0.3382	0.3365	0.3189	0.2881	0.2811	0.2642	0.3230	0.3434	0.3205	0.3384	0.0699	0.1055	0.6015	5
WL	0.3267	0.3374	0.3256	0.3249	0.3380	0.2856	0.3456	0.2481	0.3034	0.3180	0.3309	0.3757	0.4713	0.3518	0.3545	0.3614	0.3496	0.4056	0.3387	0.3618	0.0401	0.1421	0.7798	1
BN	0.3234	0.3262	0.3348	0.3051	0.3294	0.3240	0.3172	0.2959	0.3378	0.2985	0.2890	0.3056	0.3584	0.3025	0.3204	0.2904	0.3214	0.3231	0.3198	0.3460	0.0616	0.1139	0.6491	2
BS	0.2950	0.2846	0.2840	0.3535	0.2817	0.3191	0.2834	0.3875	0.3266	0.2969	0.4645	0.2580	0.2510	0.2042	0.2050	0.1785	0.1913	0.1897	0.2083	0.2565	0.1156	0.0810	0.4120	9
BM	0.3090	0.3055	0.3140	0.3305	0.2958	0.3226	0.3001	0.3360	0.3247	0.3005	0.2400	0.3124	0.2962	0.2685	0.2596	0.2391	0.2694	0.2910	0.2672	0.2889	0.0859	0.0909	0.5142	7
BL	0.3173	0.3190	0.3348	0.2972	0.3193	0.3264	0.3095	0.3099	0.3296	0.3028	0.2062	0.3798	0.3398	0.3216	0.3143	0.3055	0.3075	0.3346	0.3107	0.3042	0.0671	0.1105	0.6222	4
SM	0.2822	0.2583	0.2772	0.2734	0.2927	0.3105	0.2881	0.3707	0.2909	0.2976	0.3147	0.3139	0.2728	0.4912	0.4514	0.4639	0.4310	0.2693	0.4285	0.1887	0.0908	0.1084	0.5441	6
WR	0.2618	0.2524	0.2681	0.2790	0.2872	0.2911	0.2895	0.3160	0.2994	0.3013	0.2642	0.2599	0.2095	0.2163	0.2832	0.3000	0.2595	0.2376	0.2609	0.2563	0.1166	0.0630	0.3509	10
CK	0.2527	0.2551	0.2342	0.2370	0.2136	0.2344	0.1956	0.1865	0.1873	0.2562	0.0633	0.0796	0.1637	0.1584	0.1973	0.1680	0.1774	0.1947	0.1746	0.2478	0.1628	0.0137	0.0775	11
${\text{S}}^{+}$	0.3312	0.3439	0.3368	0.3535	0.3380	0.3264	0.3600	0.3875	0.3378	0.3180	0.4645	0.3798	0.4713	0.4912	0.4514	0.4639	0.4310	0.4056	0.4285	0.3874	—	—	—	—
S^-^	0.2527	0.2524	0.2342	0.2370	0.2136	0.2344	0.1956	0.1865	0.1873	0.2562	0.0633	0.0796	0.1637	0.1584	0.1973	0.1680	0.1774	0.1897	0.1746	0.1887	—	—	—	—

*S* ^+^ is the ideal solution, *S* ^−^ is the inverse ideal solution; *D* ^+^ is the distance of each treatment from the ideal solution; *D* ^−^ is the distance of each treatment from the inverse ideal solution.

### 3.3 Effects of different moisture-maintaining measures on garlic quality

Soil moisture-maintaining measures could improve garlic bulb quality, but the increase rate was slightly different among different measures (**Table 9**). Compared with CK, each water maintaining measure could increase the content of bulb allicin, soluble sugar, soluble protein, vitamin C, and crude fiber by 1.96% to 17.82%, 4.24% to 46.27%, 12.10% to 44.21%, 6.92% to 60.75%, and 2.97% to 31.68%, respectively, among which WL significantly increased the content of allicin, soluble protein, and crude fiber; WN significantly increased the content of soluble sugar; BL significantly increased the content of vitamin C. Although the use of moistur-maintaining measures increased the ash content and amino acid content of garlic, the increases were small and did not show significant differences with CK. Overall, the quality of garlic under transparent plastic film mulching was higher than that of black plastic film mulching, and, between oxo-biodegradable plastic film of the same color, the quality content of garlic increased with the prolongation of the induction period.

**Table 9:** Effect of different moisture-maintaining measures on the quality of purple garlic.

Year	Treatment	Allicin (mg g^−1^)	Soluble sugar (%)	Soluble protein (mg g^−1^)	Vitamin C (mg kg^−1^)	Crude fiber (%)	Ash content (%)	Amino acid (mg g^−1^)
2020	WN	7.05 ± 0.18abc	22.03 ± 0.70a	23.38 ± 0.77b	371.28 ± 13.95bc	1.32 ± 0.03ab	0.965 ± 0.03a	48.42 ± 0.93a
	WS	6.48 ± 0.07def	17.06 ± 0.43d	21.34 ± 0.53cd	310.08 ± 9.37ef	1.18 ± 0.01ef	0.921 ± 0.02a	47.41 ± 0.67a
	WM	6.81 ± 0.08cd	19.11 ± 0.70c	24.58 ± 0.46ab	406.47 ± 10.48ab	1.26 ± 0.03cd	0.937 ± 0.03a	48.63 ± 1.34a
	WL	7.34 ± 0.09a	21.94 ± 0.37a	26.52 ± 0.65a	425.34 ± 17.02a	1.36 ± 0.02a	0.945 ± 0.02a	48.82 ± 1.20a
	BN	6.97 ± 0.16bc	19.56 ± 0.53bc	23.16 ± 0.82bc	379.44 ± 12.95bc	1.30 ± 0.01bc	0.950 ± 0.02a	48.31 ± 0.92a
	BS	6.36 ± 0.07ef	16.00 ± 0.41de	20.71 ± 0.30d	291.72 ± 11.57fg	1.14 ± 0.01fg	0.924 ± 0.03a	47.21 ± 1.57a
	BM	6.72 ± 0.04cd	17.44 ± 0.53d	24.57 ± 0.49ab	388.11 ± 7.83b	1.24 ± 0.01d	0.933 ± 0.02a	48.57 ± 0.65a
	BL	7.22 ± 0.20ab	20.82 ± 0.55ab	26.45 ± 0.53a	438.60 ± 7.14a	1.35 ± 0.02ab	0.941 ± 0.02a	48.75 ± 1.67a
	SM	7.18 ± 0.05ab	19.52 ± 0.48bc	25.87 ± 0.70a	347.31 ± 12.64cd	1.21 ± 0.02de	0.928 ± 0.01a	47.88 ± 1.66a
	WR	6.60 ± 0.03de	16.17 ± 0.39de	22.81 ± 0.77bc	328.95 ± 7.52de	1.09 ± 0.02gh	0.919 ± 0.03a	47.05 ± 1.64a
	CK	6.23 ± 0.07f	15.09 ± 0.47e	18.39 ± 0.47e	272.85 ± 10.07g	1.05 ± 0.01h	0.916 ± 0.02a	46.67 ± 1.62a
2021	WN	6.85 ± 0.35ab	21.75 ± 0.65a	23.03 ± 0.80b	354.45 ± 16.01cd	1.27 ± 0.03abc	0.963 ± 0.03a	48.33 ± 1.87a
	WS	6.31 ± 0.31ab	16.75 ± 0.41c	20.95 ± 0.76cd	295.29 ± 9.85fg	1.11 ± 0.02ef	0.916 ± 0.02a	47.35 ± 1.37a
	WM	6.68 ± 0.21ab	18.74 ± 0.42b	24.47 ± 0.71ab	399.84 ± 12.52ab	1.21 ± 0.02cd	0.932 ± 0.01a	48.58 ± 0.63a
	WL	7.09 ± 0.33a	21.04 ± 0.65a	26.30 ± 0.61a	412.59 ± 11.31ab	1.33 ± 0.02a	0.940 ± 0.01a	48.76 ± 0.61a
	BN	6.77 ± 0.32ab	19.38 ± 0.35b	22.89 ± 0.74bc	357.51 ± 5.44cd	1.25 ± 0.02bc	0.946 ± 0.03a	48.21 ± 1.34a
	BS	6.23 ± 0.28ab	15.50 ± 0.53cd	20.47 ± 0.72d	287.13 ± 10.94fg	1.07 ± 0.03fg	0.920 ± 0.03a	47.15 ± 1.48a
	BM	6.56 ± 0.29ab	16.92 ± 0.51c	24.24 ± 0.41ab	385.05 ± 10.91bc	1.17 ± 0.02de	0.929 ± 0.01a	48.54 ± 0.47a
	BL	7.01 ± 0.38ab	20.25 ± 0.66ab	26.17 ± 0.32a	421.77 ± 13.67a	1.29 ± 0.02ab	0.936 ± 0.02a	48.70 ± 1.56a
	SM	6.93 ± 0.20ab	19.41 ± 0.44b	25.82 ± 0.74a	330.48 ± 10.11de	1.14 ± 0.02e	0.925 ± 0.04a	47.76 ± 1.42a
	WR	6.44 ± 0.05ab	15.80 ± 0.48cd	22.60 ± 0.68bc	307.02 ± 5.29ef	1.04 ± 0.01g	0.915 ± 0.03a	47.01 ± 0.81a
	CK	6.11 ± 0.28b	14.87 ± 0.50d	18.26 ± 0.53e	267.24 ± 9.04g	1.01 ± 0.03g	0.907 ± 0.02a	46.69 ± 0.69a
2 years average	WN	6.95 ± 0.09abc	21.89 ± 0.67a	23.21 ± 0.79c	362.87 ± 14.24cd	1.30 ± 0.03abc	0.964 ± 0.03a	48.37 ± 1.39
	WS	6.40 ± 0.12fgh	16.91 ± 0.42d	21.15 ± 0.38de	302.69 ± 9.61f	1.15 ± 0.01ef	0.919 ± 0.02a	47.38 ± 1.00
	WM	6.74 ± 0.07cde	18.93 ± 0.56c	24.53 ± 0.58bc	403.16 ± 11.49ab	1.24 ± 0.02cd	0.935 ± 0.02a	48.60 ± 0.99
	WL	7.22 ± 0.13a	21.49 ± 0.41a	26.41 ± 0.62a	418.97 ± 14.13a	1.35 ± 0.02a	0.943 ± 0.02a	48.79 ± 0.90
	BN	6.87 ± 0.08bcd	19.47 ± 0.44bc	23.03 ± 0.78c	368.48 ± 8.90cd	1.28 ± 0.01bc	0.948 ± 0.02a	48.26 ± 1.13
	BS	6.29 ± 0.11gh	15.75 ± 0.28de	20.59 ± 0.45e	289.43 ± 11.14fg	1.11 ± 0.02fg	0.922 ± 0.03a	47.18 ± 1.22
	BM	6.64 ± 0.12def	17.18 ± 0.48d	24.41 ± 0.45bc	386.58 ± 8.53bc	1.21 ± 0.02de	0.931 ± 0.02a	48.56 ± 0.56
	BL	7.11 ± 0.09ab	20.54 ± 0.42ab	26.31 ± 0.41a	430.19 ± 7.77a	1.32 ± 0.02ab	0.939 ± 0.02a	48.72 ± 1.52
	SM	7.05 ± 0.07ab	19.46 ± 0.46bc	25.85 ± 0.58ab	338.90 ± 6.52de	1.18 ± 0.01e	0.927 ± 0.02a	47.82 ± 1.42
	WR	6.52 ± 0.01efg	15.99 ± 0.35de	22.71 ± 0.61cd	317.99 ± 5.61ef	1.07 ± 0.02gh	0.917 ± 0.02a	47.03 ± 1.22
	CK	6.17 ± 0.11h	14.98 ± 0.46e	18.33 ± 0.46f	270.05 ± 8.04g	1.03 ± 0.02h	0.912 ± 0.02a	46.68 ± 1.13
ANOVA								
Year (Y)		ns	ns	ns	**	***	ns	ns
Treatment (T)		***	***	***	***	***	ns	ns
Y × T		ns	ns	ns	ns	ns	ns	ns

Different lowercase letters in the same column indicate significant differences among different treatments (*P* < 0.05). The ** and *** indicate significant differences among different treatments at the levels of *P* < 0.01 and *P* < 0.001, respectively. The ns means not significant at the level of *P* ≥ 0.05.

### 3.4 Effects of different moisture-maintaining measures on garlic yield

The use of moisture-maintaining measures to increase garlic yield also increased water–fertilizer productivity, and the increases varied between measures (**Table 10**). The WR had the lowest yield, which increased only 489.14 to 1,082.55 kg hm^−2^ compared with that of CK, and there was no significant difference; whereas the other treatments showed significant advantages in increasing yield, which could be 32.70% to 158.96%, with the highest yield of WN and WL, which could be 181,93.09 to 18,661.33 kg hm^−2^ and 18,064.73 to 18,796.05 kg hm^−2^, respectively, which were significantly increased by 128.27% to 158.96% and 129.92% to 157.13%, respectively, compared with that of CK. The use of moisture-maintaining measures could increase the water productivity of garlic, in which the WN had the highest water productivity of 3.10 to 3.14 kg m^−3^, followed by the WL and BN, which significantly increased by 145.31% to 164.96%, 141.41% to 157.26%, and 114.06% to 123.93% compared with that of CK, respectively. The water productivity of WR was the least, which was only 6.25% to 13.68% higher than that of CK, and there was no significant difference. Irrigation water productivity and nitrogen, potassium, and phosphate fertilizer partial factor productivity performance trends were consistent with yield change; WN and WL were the highest; WR was the lowest; and there was no significant difference from CK.

**Table 10:** Effect of different moisture-maintaining measures on yield and water productivity of purple garlic.

Year	Treatment	Yield (kg hm^−2^)	Water productivity (kg m^−3^)	Irrigation water productivity (kg m^−3^)	N fertilizer partial factor productivity (kg kg^−1^)	P fertilizer partial factor productivity (kg kg^−1^)	K fertilizer partial factor productivity (kg kg^−1^)
2020	WN	18,661.33 ± 751.45a	3.14 ± 0.14a	4.20 ± 0.17a	83.31 ± 3.35a	119.62 ± 4.82a	82.94 ± 3.34a
	WS	14,071.49 ± 708.61d	2.21 ± 0.10d	3.17 ± 0.16d	62.82 ± 3.16d	90.20 ± 4.54d	62.54 ± 3.15d
	WM	17,063.28 ± 833.20ab	2.77 ± 0.14b	3.84 ± 0.19ab	76.18 ± 3.72ab	109.38 ± 5.34ab	75.84 ± 3.70ab
	WL	18,796.05 ± 801.97a	3.09 ± 0.15a	4.23 ± 0.18a	83.91 ± 3.58a	120.49 ± 5.14a	83.54 ± 3.57a
	BN	16327.94 ± 476.01bc	2.74 ± 0.05b	3.68 ± 0.11bc	72.89 ± 2.12bc	104.67 ± 3.05bc	72.57 ± 2.12bc
	BS	13,761.57 ± 289.72d	2.18 ± 0.03d	3.10 ± 0.07d	61.44 ± 1.29d	88.22 ± 1.86d	61.16 ± 1.29d
	BM	14,479.16 ± 730.34cd	2.36 ± 0.10cd	3.26 ± 0.17cd	64.64 ± 3.26cd	92.82 ± 4.68cd	64.35 ± 3.25cd
	BL	15,953.72 ± 749.07bc	2.61 ± 0.11bc	3.59 ± 0.17bc	71.22 ± 3.34bc	102.27 ± 4.80bc	70.91 ± 3.33bc
	SM	10,848.65 ± 299.52e	1.72 ± 0.03e	2.44 ± 0.07e	48.43 ± 1.34e	69.54 ± 1.92e	48.22 ± 1.33e
	WR	8,664.23 ± 290.27f	1.36 ± 0.05f	1.95 ± 0.07f	38.68 ± 1.30f	55.54 ± 1.86f	38.51 ± 1.29f
	CK	8,175.09 ± 215.77f	1.28 ± 0.03f	1.84 ± 0.05f	36.50 ± 0.96f	52.40 ± 1.38f	36.33 ± 0.96f
2021	WN	18,193.09 ± 977.36a	3.10 ± 0.16a	4.52 ± 0.24a	81.22 ± 4.36a	116.62 ± 6.27a	80.86 ± 4.34a
	WS	13,415.83 ± 626.27c	2.22 ± 0.09d	3.33 ± 0.16c	59.89 ± 2.80c	86.00 ± 4.01c	59.63 ± 2.78c
	WM	15,460.94 ± 264.12b	2.50 ± 0.02bc	3.84 ± 0.07b	69.02 ± 1.18b	99.11 ± 1.69b	68.72 ± 1.17b
	WL	18,064.73 ± 176.28a	3.01 ± 0.02a	4.48 ± 0.04a	80.65 ± 0.79a	115.80 ± 1.13a	80.29 ± 0.78a
	BN	15,767.05 ± 642.92b	2.62 ± 0.09b	3.91 ± 0.16b	70.39 ± 2.87b	101.07 ± 4.12b	70.08 ± 2.86b
	BS	12904.17 ± 591.07cd	2.17 ± 0.10d	3.20 ± 0.15cd	57.61 ± 2.64cd	82.72 ± 3.79cd	57.35 ± 2.63cd
	BM	13,832.67 ± 511.83c	2.30 ± 0.09cd	3.43 ± 0.13c	61.75 ± 2.28c	88.67 ± 3.28c	61.48 ± 2.27c
	BL	15,610.42 ± 620.59b	2.56 ± 0.10bc	3.88 ± 0.15b	69.69 ± 2.77b	100.07 ± 3.98b	69.38 ± 2.76b
	SM	11,351.38 ± 514.60d	1.88 ± 0.09e	2.82 ± 0.13d	50.68 ± 2.30d	72.77 ± 3.30d	50.45 ± 2.29d
	WR	8,107.96 ± 144.75e	1.33 ± 0.03f	2.01 ± 0.04e	36.20 ± 0.65e	51.97 ± 0.93e	36.04 ± 0.64e
	CK	7,025.41 ± 253.48e	1.17 ± 0.05f	1.74 ± 0.06e	31.36 ± 1.13e	45.03 ± 1.62e	31.22 ± 1.13e
2 years average	WN	18,427.21 ± 853.86a	3.12 ± 0.15a	4.36 ± 0.20a	82.27 ± 3.81a	118.12 ± 5.47a	81.90 ± 3.80a
	WS	13,743.66 ± 609.62c	2.22 ± 0.09c	3.25 ± 0.14c	61.36 ± 2.72c	88.10 ± 3.91c	61.09 ± 2.71c
	WM	16,262.11 ± 545.78b	2.64 ± 0.08b	3.84 ± 0.13b	72.60 ± 2.44b	104.25 ± 3.50b	72.28 ± 2.43b
	WL	18,430.39 ± 476.88a	3.05 ± 0.07a	4.36 ± 0.11a	82.28 ± 2.13a	118.15 ± 3.06a	81.92 ± 2.12a
	BN	16,047.50 ± 554.80b	2.68 ± 0.08b	3.80 ± 0.13b	71.64 ± 2.48b	102.87 ± 3.55b	71.33 ± 2.47b
	BS	13,332.87 ± 412.54c	2.18 ± 0.09c	3.15 ± 0.10c	59.53 ± 1.84c	85.47 ± 2.64c	59.26 ± 1.83c
	BM	14,155.92 ± 600.05c	2.33 ± 0.10c	3.35 ± 0.14c	63.20 ± 2.68c	90.75 ± 3.85c	62.92 ± 2.67c
	BL	15,782.07 ± 647.09b	2.59 ± 0.10b	3.74 ± 0.15b	70.46 ± 2.89b	101.17 ± 4.15b	70.15 ± 2.88b
	SM	11,100.02 ± 406.96d	1.80 ± 0.06d	2.63 ± 0.10d	49.56 ± 1.82d	71.16 ± 2.62d	49.34 ± 1.81d
	WR	8,386.10 ± 195.66e	1.35 ± 0.03e	1.98 ± 0.05e	37.44 ± 0.87e	53.76 ± 1.25e	37.28 ± 0.87e
	CK	7,600.25 ± 213.77e	1.23 ± 0.04e	1.79 ± 0.05e	33.93 ± 0.95e	48.72 ± 1.37e	33.78 ± 0.95e
ANOVA							
Year (Y)		*	ns	**	*	*	*
Treatment (T)		***	***	***	***	***	***
Y × T		ns	ns	ns	ns	ns	ns

Different lowercase letters in the same column indicate significant differences among different treatments (*P* < 0.05). The *, **, and *** indicate significant differences among different treatments at the levels of *P* < 0.05, *P* < 0.01, and *P* < 0.001, respectively. The ns means not significant at the level of *P* ≥ 0.05.

### 3.5 Principal component analysis

Interactions between factors affect the growth of purple garlic, which makes many index information interweave and overlap. Principal component analysis (PCA) is a statistical analysis method that transforms multiple indices into fewer comprehensive indices to reveal the internal structure among multiple variables with less information loss (Hu et al., 2019). The application of PCA can be used to sift out a number of comprehensive indices that are not related to each other in a complex quality indices and growth indices and can reflect most of the information provided by the original full set of indices (Zhang et al., 2023a). In this study, a total of 13 data on yield, water productivity, and bulb quality indices of purple garlic were selected for PCA, and six principal components were finally generated. As can be seen from **Table 11**, the six principal components generated eigenvalues of 10.94, 1.33, 0.48, 0.15, 0.06, and 0.02, respectively, and their contribution rates to the total variance was 84.18%, 10.25%, 3.66%, 1.15%, 0.45%, and 0.17%, respectively. According to the principle that the cumulative contribution rate of the number of extracted principal components is ≥85%, the cumulative contribution rate of the six principal components has reached 99.86%, indicating that yield, water productivity, nitrogen fertilizer partial factor productivity, allicin, amino acids, and soluble sugar can measure all the information of these 13 indices, and, therefore, only these six indices were selected to establish the comprehensive evaluation equation.

**Table 11:** Principal component analysis of indices affecting the growth of purple garlic.

Serial number	Index	Principal component 1	Principal component 2	Principal component 3	Principal component 4	Principal component 5	Principal component 6
1	Yield	0.2893	−0.2443	−0.0826	0.1110	0.0453	0.0138
2	Water productivity	0.2893	−0.2495	−0.0290	0.0568	0.0576	0.0138
3	N fertilizer partial factor productivity	0.2893	−0.2443	−0.0826	0.1110	0.0494	0.0138
4	Allicin	0.2524	0.4306	0.2812	0.2995	−0.1194	0.2553
5	Amino acid	0.2835	0.1707	−0.3145	−0.3770	0.0494	−0.5935
6	Soluble sugar	0.2778	0.1672	0.4363	0.2427	−0.4240	−0.5659
7	Soluble protein	0.2246	0.5407	−0.2015	0.3202	0.6134	0.0207
8	Vitamin C	0.2621	0.3145	−0.3971	−0.4389	−0.3376	0.2070
9	Crude fiber	0.2984	0.0788	0.0319	−0.0594	−0.3623	0.4761
10	Ash content	0.2615	−0.0260	0.6305	−0.5861	0.4076	0.1035
11	Irrigation water productivity	0.2896	−0.2417	−0.0812	0.1110	0.0535	0.0276
12	P fertilizer partial factor productivity	0.2893	−0.2435	−0.0841	0.1110	0.0453	0.0138
13	K fertilizer partial factor productivity	0.2893	−0.2435	−0.0826	0.1110	0.0453	0.0138
Eigenvalue		10.94	1.33	0.48	0.15	0.06	0.02
Contribution rate		84.18	10.25	3.66	1.15	0.45	0.17
Accumulate contribution rate		84.18	94.43	98.09	99.24	99.70	99.86

### 3.6 Evaluation of various moisture-maintaining measures based on PCA

Because the 13 indices in this test had different scales, in order to eliminate the influence of scales and orders of magnitude on the evaluation results, it is necessary to standardize the original data to ensure the objectivity and scientificity of the PCA results. The standardized indices were represented by X_1_~X_14_ respectively, and the comprehensive evaluation equation for each principal component is as follows:


$$\begin{array}{c} Y_{1}=0.2893X_{1}+0.2893X_{2}+0.2893X_{3}+0.2524X_{4}+0.2835X_{5}\\ +0.2778X_{6}+0.2246X_{7}+0.2621X_{8}+0.2984X_{9}+0.2615X_{10}\\ +0.2896X_{11}+0.2893X_{12}+0.2893X_{13} \end{array}$$

$$\begin{array}{c} Y_{2}=-0.2443X_{1}-0.2495X_{2}-0.2443X_{3}+0.4306X_{4}+0.1707X_{5}\\ +0.1672X_{6}+0.5407X_{7}+0.3145X_{8}+0.0788X_{9}-0.0260X_{10}\\ -0.2417X_{11}-0.2435X_{12}-0.2435X_{13} \end{array}$$

$$\begin{array}{c} Y_{3}=-0.0826X_{1}-0.0290X_{2}-0.0826X_{3}+0.2812X_{4}-0.3145X_{5}\\ +0.4363X_{6}-0.2015X_{7}-0.3971X_{8}+0.0319X_{9}+0.6305X_{10}\\ -0.0812X_{11}-0.0841X_{12}-0.0826X_{13} \end{array}$$

$$\begin{array}{c} Y_{4}=0.1110X_{1}+0.0568X_{2}+0.1110X_{3}+0.2995X_{4}-0.3770X_{5}\\ +0.2427X_{6}+0.3202X_{7}-0.4389X_{8}-0.0594X_{9}-0.5861X_{10}\\ +0.1110X_{11}+0.1110X_{12}+0.1110X_{13} \end{array}$$

$$\begin{array}{c} Y_{5}=0.0453X_{1}+0.0576X_{2}+0.0494X_{3}-0.1194X_{4}+0.0494X_{5}\\ -0.4240X_{6}+0.6134X_{7}-0.3376X_{8}-0.3623X_{9}+0.4076X_{10}\\ +0.0535X_{11}+0.0453X_{12}+0.0453X_{13} \end{array}$$

$$\begin{array}{c} Y_{6}=0.0138X_{1}+0.0138X_{2}+0.0138X_{3}+0.2553X_{4}-0.5935X_{5}\\ -0.5659X_{6}+0.0207X_{7}+0.2070X_{8}+0.4761X_{9}+0.1035X_{10}\\ +0.0276X_{11}+0.0138X_{12}+0.0138X_{13} \end{array}$$

Ranking by the D value of the comprehensive evaluation of the affiliation function was WL>WN>BL>BN>WM>BM>SM>WS>BS>WR>CK (**Table 12**), indicating that each moisture-maintaining measure could significantly increase garlic yield, bulb quality, and water–fertilizer productivity compared to the traditional planting system, in which the use of 110*-*day transparent oxo-biodegradable plastic film mulching was the most effective.

**Table 12:** Comprehensive evaluation of the effects of different treatments on garlic yield, water–fertilizer productivity, and bulb quality indices.

Treatment	F1	F2	F3	F4	F5	F6	U1	U2	U3	U4	U5	U6	D	Sequence
WN	3.7008	−1.1608	1.2700	−0.1721	0.0705	−0.2023	0.9405	0.1329	1.0000	0.3401	0.7162	0.0209	0.8504	2
WS	−1.5272	−1.3152	−0.2538	0.4570	−0.0252	0.0187	0.4336	0.0909	0.3228	0.9412	0.5924	0.5664	0.4012	8
WM	1.8169	−0.1360	−0.9712	−0.0735	−0.0639	−0.2107	0.7578	0.4121	0.0040	0.4343	0.5423	0.0000	0.6887	5
WL	4.3145	0.3428	−0.2646	0.5185	−0.2364	0.0853	1.0000	0.5425	0.3180	1.0000	0.3192	0.7306	0.9245	1
BN	1.9516	−0.4874	0.6371	−0.4919	0.0372	0.1944	0.7709	0.3163	0.7188	0.0345	0.6730	1.0000	0.7138	4
BS	−2.2251	−1.6488	−0.2929	0.3378	0.2598	0.1236	0.3659	0.0000	0.3055	0.8273	0.9611	0.8253	0.3349	9
BM	0.3748	0.2891	−0.9802	−0.5280	0.2899	−0.0921	0.6180	0.5278	0.0000	0.0000	1.0000	0.2928	0.5801	6
BL	2.7987	1.2123	−0.2607	−0.1682	−0.2415	0.1217	0.8530	0.7793	0.3198	0.3438	0.3127	0.8204	0.8175	3
SM	−0.9858	2.0226	0.6705	0.4948	0.1957	−0.1412	0.4861	1.0000	0.7336	0.9774	0.8781	0.1715	0.5548	7
WR	−4.2203	1.1851	0.2069	−0.1378	0.1971	0.1679	0.1724	0.7719	0.5276	0.3728	0.8799	0.9346	0.2537	10
CK	−5.9988	−0.3037	0.2389	−0.2365	−0.4832	−0.0653	0.0000	0.3664	0.5418	0.2785	0.0000	0.3590	0.0613	11

### 3.7 Correlation analysis between soil quality indices and growth indices of purple garlic

**Figure 10:**
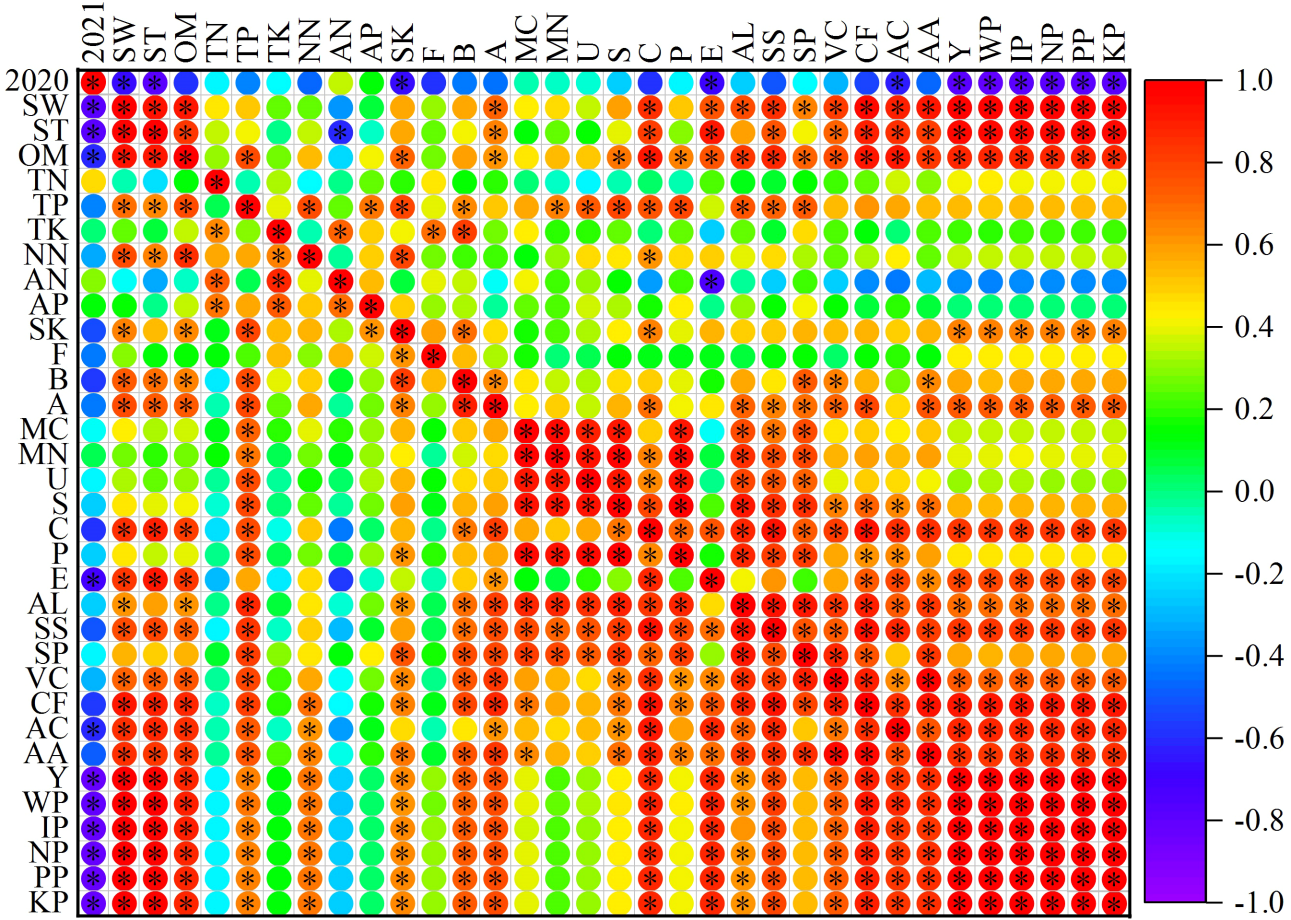
Pearson’s correlation analyses between yield and water productivity and nitrogen use efficiency under drip irrigation under plastic film mulching in growth periods of purple garlic in 2020 and 2021. Correlations between two indices were significant at *p < 0.01. SW, soil water storage; ST, soil temperature; OM, organic matter; TN, total nitrogen; TP, total phosphorus; TK, total potassium; NN, nitrate nitrogen; AN, ammonium nitrogen; AP, available phosphorus; SK, slow-release potassium; F, fungi; B, bacterium; A, actinobacteria; MC, soil microbial biomass carbon; MN, soil microbial biomass nitrogen; U, urease; S, sucrase; C, catalase; P, alkaline phosphatase; E, cellulase; AL, allicin; SS, soluble sugar; SP, soluble protein; VC, vitamin C; CF, crude fiber; AC, ash content; AA, amino acid; Y, yield; WP, water productivity; IP, irrigation water productivity; NP, nitrogen fertilizer partial factor productivity; PP, potassium fertilizer partial factor productivity; KP, phosphate fertilizer partial factor productivity.

As can be seen from **Figure 10**, there were significant positive correlations between soil water storage, soil temperature, organic matter, actinobacteria, catalase, cellulase, yield, quality indices, and water–fertilizer productivity, and there was also a significant positive correlation between soil water storage and soil temperature but no significant correlation with other indicators, which indicated that, with the improvement of the soil hydrothermal environment conditions, the content of soil organic matter and the number of actinobacteria increased and that the activities of catalase and cellulase were increased, which led to a significant increase in the quality, yield, and water–fertilizer productivity of garlic. Soil microbial biomass carbon and nitrogen were significantly positively correlated not only with the activities of urease, sucrase, and alkaline phosphatase but also with allicin, soluble sugar, and soluble protein. All these results indicated that microbial biomass had an important influence on the activities of soil urease, sucrase, and alkaline phosphatase and significantly contributed to the accumulation of allicin, soluble sugar, and soluble protein. Allicin, soluble sugar, vitamin C, crude fiber, ash content, and amino acid were significantly positively correlated with each other, whereas soluble protein was significantly positively correlated with vitamin C, crude fiber, and amino acid, suggesting that garlic quality indices can be both mutually reinforcing and independent of each other, and integrally affect quality. There was a significant positive correlation between garlic yield and water–fertilizer productivity, that is, an increase in yield was accompanied by a corresponding increase in water–fertilizer productivity.

### 3.8 Path analysis of soil physical and chemical properties on yield and quality of purple garlic

**Figure 11:**
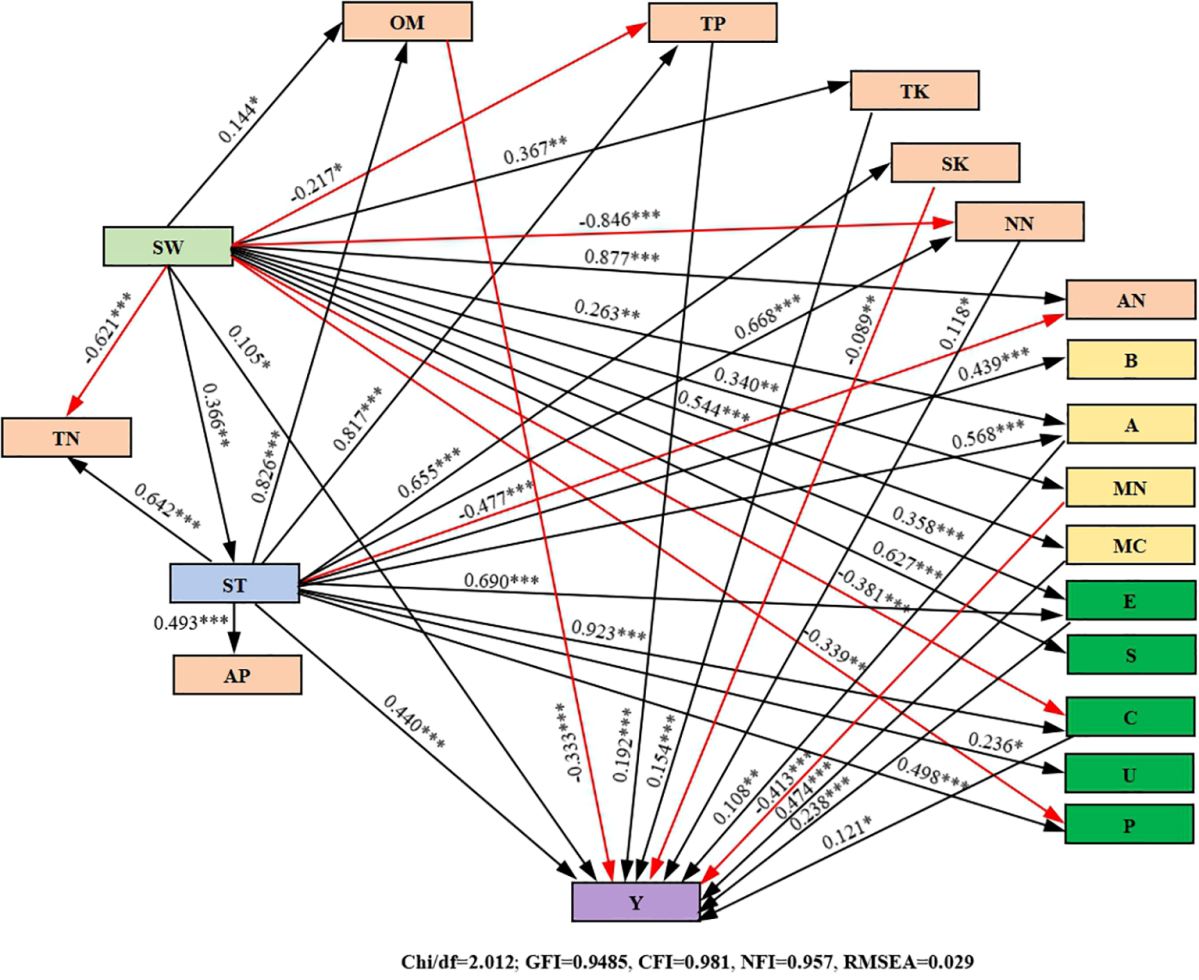
Path analysis of soil physical and chemical properties on yield of purple garlic. The data on the arrows are standardized path coefficients (λ). The black arrows indicate positive impacts. The red arrows indicate negative impacts. SW, soil water storage; ST, soil temperature; OM, organic matter; TN, total nitrogen; TP, total phosphorus; TK, total potassium; NN, nitrate nitrogen; AN, ammonium nitrogen; AP, available phosphorus; SK, slow-release potassium; B, bacterium; A, actinobacteria; MC, soil microbial biomass carbon; MN, soil microbial biomass nitrogen; U, urease; S, sucrase; C, catalase; P, alkaline phosphatase; E, cellulase; Y, yield. The *, **, and *** indicate significant correlation of different indicators at the levels of P < 0.05, P < 0.01, and P < 0.001, respectively.

**Figure 12:**
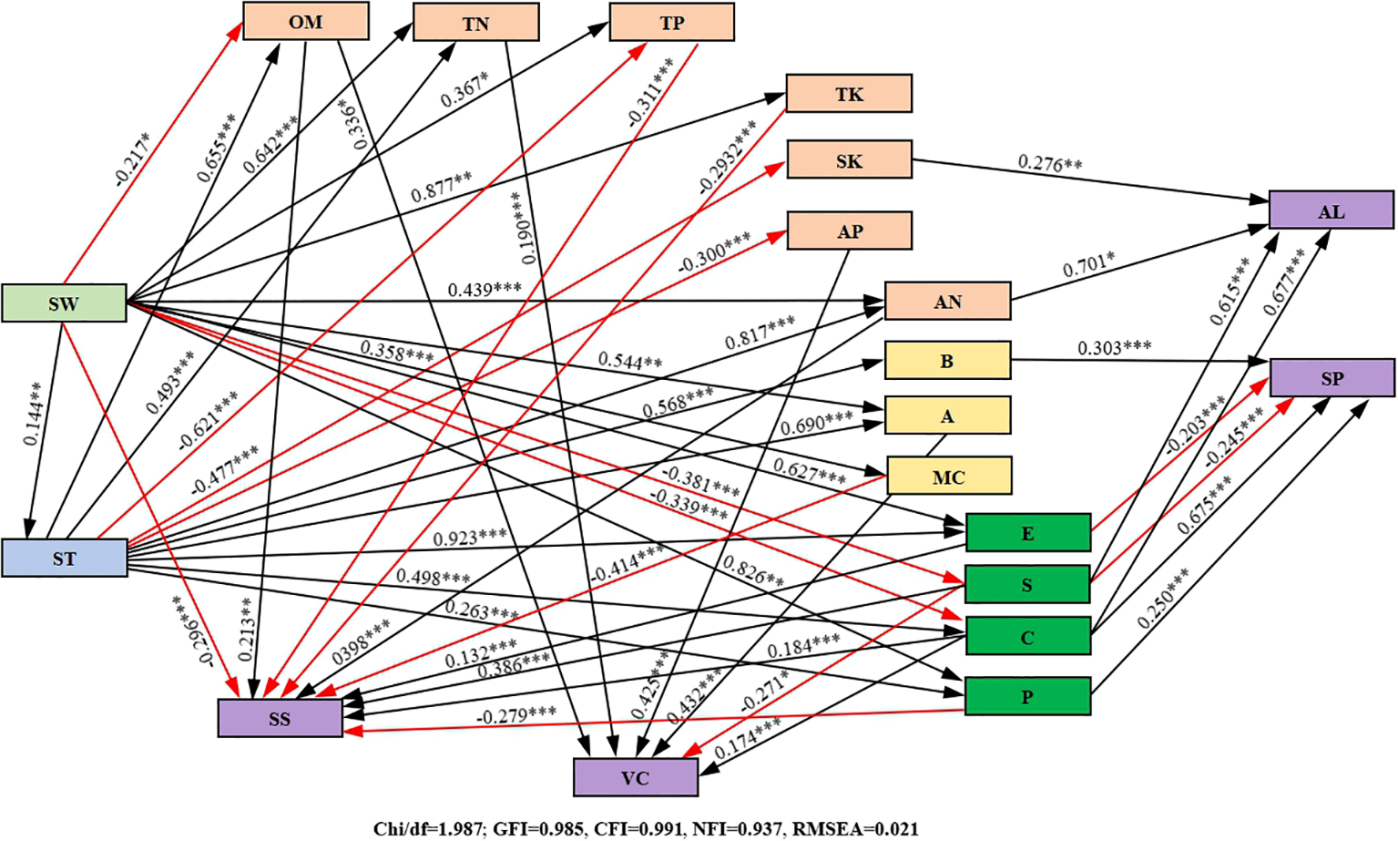
Path analysis of soil physical and chemical properties on quality of purple garlic. The data on the arrows are standardized path coefficients (λ). The black arrows indicate positive impacts. The red arrows indicate negative impacts. SW, soil water storage; ST, soil temperature; OM, organic matter; TN, total nitrogen; TP, total phosphorus; TK, total potassium; AN, ammonium nitrogen; AP, available phosphorus; SK, slow-release potassium; B, bacterium; A, actinobacteria; MC, soil microbial biomass carbon; S, sucrase; C, catalase; P, alkaline phosphatase; E, cellulase; AL, allicin; SS, soluble sugar; SP, soluble protein; VC, vitamin C. The *, **, and *** indicate significant correlation of different indicators at the levels of P < 0.05, P < 0.01, and P < 0.001, respectively.

As can be seen in **Figure 11**, soil water storage, soil temperature, soil nutrients, soil microorganisms, and soil enzyme activities have direct or indirect effects with the yield of purple garlic. The degree of influence of soil factors on the yield of purple garlic was in the order of soil microbial biomass carbon > soil temperature > cellulase > total phosphorus > total potassium > catalase > nitrate nitrogen > actinobacteria > soil water storage. Soil microbial biomass carbon and soil temperature were important factors promoting the increase in yield of purple garlic, whereas soil microbial biomass nitrogen, organic matter, and slow-release potassium were the factors inhibiting the increase in yield of purple garlic. As can be seen in **Figure 12**, soil water storage, soil temperature, soil nutrients, soil microorganisms, and soil enzyme activities also have direct or indirect effects with the quality of purple garlic. Among them, the degree of influence of soil factors on allicin of purple garlic was in the order of ammonium nitrogen > catalase > sucrase > slow-release potassium. The degree of effect on soluble protein of purple garlic was in the following order: catalase > bacterial > alkaline phosphatase, whereas sucrase and cellulase were the factors that inhibited the increase of soluble protein of purple garlic. The degree of influence on soluble sugars of purple garlic was in the order of ammonium nitrogen > sucrase > organic matter > catalase > cellulase, whereas soil water storage, total phosphorus, total potassium, soil microbial biomass carbon, and alkaline phosphatase were the factors inhibiting the increase of soluble sugars of purple garlic. The degree of influence on vitamin C of purple garlic was in the order of actinobacteria > available phosphorus > organic matter > total nitrogen > catalase, whereas sucrase was the factor inhibiting the increase of vitamin C of purple garlic.

## 4 Discussion

### 4.1 Effect of soil moisture-maintaining measures on soil water storage characteristics

The soil moisture intense evaporation during the growing period of purple garlic in the China Hexi Corridor oasis agricultural area and the precipitation are very limited, with large inter-annual and intra-annual differences in precipitation, especially during the sowing stage; there is virtually no precipitation, and channel irrigation water has not been supplied; at this time, the soil water storage will be a very important source of water supply for garlic. Therefore, taking appropriate moisture-maintaining measures can effectively reduce the ineffective evaporation of water, store soil water, reduce the unproductive water consumption of farmland, optimize the soil water environment of farmland, and enhance the water use of crops. Numerous studies had confirmed that plastic film, straw mulching, and A-SAP application can effectively improve soil water conditions, which can inhibit soil water evaporation and increase water storage during the whole growth stage of crops (Tang et al., 2016; Tian et al., 2020). Surface mulching avoids evapotranspiration of most of the soil water storage during the garlic growing season, reduces ineffective water evaporation, forces the lateral migration of water, and therefore has a significant mitigating effect on soil-crop drought stress (Lin et al., 2014; Zhou et al., 2009). This study showed that, compared with the open-field planting system, both transparent plastic film and black plastic film could significantly increase the soil water storage in the 0- to 100-cm soil layer by up to 16.00% to 48.59% and 14.84% to 44.35%, respectively, and the water storage capacity of the transparent plastic film mulching was better than that of the black plastic film mulching as a whole. Oxo-biodegradable plastic film had significant moisture-maintaining effect in garlic seedling stage and scaly bud and flower bud differentiation stage, but the water storage effect gradually weakened with the expanding degree of cleavage. Therefore, WL and BL had a better moisture-maintaining effect. Compared with open-field planting, garlic harvesting can significantly increase soil water storage by 25.38% to 42.45% and 19.50% to 30.66%, whereas WM, BM, WS, and BS did not show lasting moisture-maintaining effect due to earlier induction period, and similar conclusion was also shown in the results of Wu et al. (2022). There have been many studies on the moisture-maintaining effect of straw and plastic film mulching. Li et al. (2013) showed that straw mulching improved the intensity of precipitation infiltration, so the soil moisture increased effect of straw mulching was better than that of ordinary transparent plastic film mulching and oxo-biodegradable plastic film mulching, whereas Chen et al. (2012) showed that the moisture increased effect of ordinary transparent plastic film was better than that of oxo-biodegradable plastic film and straw mulching. The results of this experiment showed that straw mulching showed moisture-maintaining advantages throughout the growth stage of garlic, and the soil water storage was increased by 7.35% to 17.20% compared with that of open-field planting as a whole, the inter-annual increase varied greatly, and the water storage capacity was lower than that of ordinary transparent plastic film and oxo-biodegradable plastic film. The results of this study better confirmed the findings of Chen et al. (2012) but were different from those conclusions of Li et al. (2013), which may be related to factors such as differences in precipitation in the experimental area and different water consumption of planted crops. A-SAP is as a new type of polymer water-absorbing material; Yang et al. (2015) concluded that the application of A-SAP can effectively improve soil water absorption and water-holding capacity, which can significantly improve the soil water status of the 0- to 60-cm soil layer, and the soil water storage continues to rise with the increase of the application of A-SAP. However, in other related studies, it was shown that excessive application of A-SAP caused excessive cohesion of soil particles and clogging of soil pores by swollen A-SAP sols, which was instead unfavorable for soil water infiltration and storage (Liu et al., 2022a). In this study, the application of 45 kg hm^−2^ A-SAP increased the soil water storage capacity of 0- to 100-cm soil layer in each growth stage of garlic by 0.77% to 9.47% compared with that of open field, but the increase was small and not significantly different from that of open field. However, the gradient of application rate was not set in this experiment, and further experiments are needed to verify whether the soil water storage capacity continued to increase with the increase of application rate.

### 4.2 Effect of soil moisture-maintaining measures on soil temperature

Although purple garlic is a shade-loving crop, unreasonable mulching measures caused the soil accumulated temperature to be too high, which exceeds the appropriate growth temperature, leading to yellowing of the stems and leaves of garlic and growth retardation. Therefore, optimal moisture-maintaining measures not only can provide adequate water but also can improve soil temperature and maintain a favorable hydrothermal environment. Different mulching measures have different effects on the regulation of soil temperature. Plastic film mulching can block the heat exchange between the soil and the external environment, and, at the same time, the inner membrane attached to the aquifer to weaken the short-wave radiation of the sun during the daytime and the long-wave radiation of the soil at night, the warming during the daytime, and the cooling at night are slowed down, so that the average temperature of the soil increased significantly (Ochege et al., 2022). Wang et al. (2021) showed that, in the early stage of crop growth, the film surface of oxo-biodegradable plastic film remained intact, and the difference between the thermal insulation effect and that of ordinary transparent plastic film was not significant, but, as the film surface of oxo-biodegradable plastic film degraded and ruptured, the soil temperature under the film began to decrease, and, in the late stage of crop growth, the average daily soil temperature of oxo-biodegradable plastic film was significantly lower than that of ordinary transparent plastic film. The results of this study showed that, compared with the open-field planting mode, plastic film mulching can significantly increase the soil temperature during the growth period of garlic. There was no significant difference in soil temperature between the plastic film of the same color because the surface of the oxo-biodegradable plastic film remained intact during the sowing period, seedling period, and scaly bud and flower bud differentiation stage of garlic. However, as the oxo-biodegradable plastic film entered the induction, cracking and macro-cracking periods, the soil temperature was significantly lower than that of ordinary plastic film, whereas the oxo-biodegradable plastic film with later induction did not show significant changes, which better verified the viewpoints of Wang et al. (2021). However, this study further found that, as garlic entered the mature stage, the effect of plastic film warming again prominent; especially, the ordinary plastic film mulching was the most significant, whereas the soil temperature of oxo-biodegradable plastic film significantly decreased compared with ordinary plastic film, which was slightly different from the results of the study by Liu et al. (2021), which is due to the different types of planted crops; the aboveground stalks of garlic gradually dried up during the mature stage; and the soil temperature increased significantly due to the direct light shining on the surface. Straw mulching has a weaker barrier to air heat compared to plastic film, and, at the same time, it can reduce the direct sunlight on the ground to a certain extent, thus weakening the influence of external environment on soil temperature (Liu et al., 2022b). The results of this study found that the straw mulching soil temperature was still significantly increased by 14.70% to 19.80% and 10.82% to 16.29% in the sowing and seedling stages of garlic than that of open field, whereas there was no significant difference in the middle and late stages of growth, and the results of this experiment better confirmed the above conclusion. There are different conclusions about the effect of A-SAP on soil temperature. Zhang et al. (2012) showed that the application of A-SAP kept more water in the soil layer, and the average daily soil temperature was reduced due to the high heat capacity of water, which impeded the conduction of solar radiation to the deeper soil layers; whereas Yang et al. (2020) showed that the application of A-SAP increased the average daily soil temperature. In this study, although the application of A-SAP reduced the soil temperature, there was no significant difference with the open field, which better supported the conclusion of Zhang et al. (2012). The reason for the difference with the research results of Yang et al. (2020) may be related to the process of A-SAP absorption and release.

### 4.3 Effect of soil moisture-maintaining measures on soil nutrient

The use of reasonable moisture-maintaining measures not only can improve the soil erosion resistance and scour resistance but also can effectively reduce soil erosion and nutrient leaching, so as to achieve the role of nutrient retention. Fu et al. (2021) found that plastic film mulching improved soil hydrothermal conditions, accelerated soil organic carbon mineralization, and increased organic carbon input of crop roots, which resulted in a significant increase in soil organic matter and fast-release nutrient content; whereas Rahmani et al. (2021) found that mulching treatments had a significant effect on soil total potassium content but not on soil organic matter, total nitrogen, and fast-release phosphorus content. Yang et al. (2022a) found that plastic film mulching could promote the growth and development of the crop roots and promote crops to absorb more nutrients, which in turn, caused a significant reduction in the soil fast-release nutrient content. The results of this experiment showed that plastic film mulching can significantly increase the soil nutrient content in the 0- to 20-cm soil layer compared with that in the open field, only the available phosphorus content in the 20- to 40-cm soil layer showed a decreasing change, and the other nutrient parameters also showed a tendency to increase. With the prolongation of oxo-biodegradable plastic film induction period, the content of soil organic matter, total phosphorus, nitrate nitrogen, and slow-release potassium showed an increasing change, whereas the content of total potassium and ammonium nitrogen showed a decreasing change, which was similar to the relevant research results of Fu et al. (2021) and also confirmed the conclusions of Duan et al. (2022), but there was a difference from the results of the studies by Rahmani et al. (2021) and Yang et al. (2022a), which may be related to the climatic environment of the experimental area; the characteristics of soil hydrothermal environment improved by plastic film mulching and the growth state of crops. The improvement of soil nutrient content by straw mulching has been confirmed in many studies (Dung et al., 2023; Lv et al., 2023), and this study is no exception, which also showed that soil nutrient content could be significantly increased by straw mulching, which was mainly due to the fact that after straw mulching; straw near the soil surface layer was gradually decomposed into organic matter in hydrothermal environment, which made the soil organic matter content rise; and, at the same time, heat was generated, which slowed down the soil nutrient loss and better maintain land productivity. Application of A-SAP can also improve soil nutrient status and enhance soil fertilizer retaining performance (He et al., 2023; Li et al., 2022c). The results of this study showed that the application of A-SAP also increased soil organic matter and soil nutrient content, and the increase in nutrient content in the 20- to 40-cm soil layer was greater than that in the 0- to 20-cm soil layer, which was in agreement with the research results of Cao et al. (2017), but Jamal et al. (2022) concluded that the application of A-SAP accelerated the transfer rate of nutrients from the soil to the plant, and, at the same time, due to the effect of fertilizer absorption and retention, which reduced the soil nutrient content, the reason for the difference in conclusions may be related to factors such as different tillage practices, A-SAP application rates, and the application depth.

### 4.4 Effect of soil moisture-maintaining measures on soil microorganisms

Soil microorganisms are involved in soil ecological functions, environmental functions, and immune functions to coordinate the regulation of soil health, which is the core and key to maintaining soil quality (Daunoras et al., 2024). Soil microorganisms with the largest populations of soil bacterium, actinobacteria, and fungi are extremely sensitive to environmental changes, and changes in soil physicochemical properties can significantly affect the population composition (Guo et al., 2018). Therefore, the selection of reasonable moisture-maintaining measures can promote reproductive growth by improving the microbial living environment such as soil hydrothermal and aeration. Many studies have shown that either plastic film mulching or straw mulching can increase the relative abundance and activity of soil flora and improve the diversity and structure of soil microbial communities (Li et al., 2022d; Liu et al., 2023), but the conclusions varied due to the different climatic conditions, soil types, crop species, fertilizer application, etc., in the experimental areas; e.g., Zhang et al. (2024) showed that plastic film mulching or straw mulching increased the number of bacterium, fungi, and actinobacteria in soil, and the effect of straw mulching was better than that of plastic film mulching; whereas Li et al. (2022a) showed that plastic film mulching could significantly increase the total number of soil microorganisms, but the number of fungi decreased, and the oxo-biodegradable plastic film has the same effect as that of ordinary plastic film. In this study, it was found that plastic film mulching or straw mulching could significantly increase the number of soil fungi, bacterium, and actinobacteria in the 0- to 40-cm soil layer of garlic farmland, which better confirmed the study conclusion of the former. This study further found that, with the prolongation of the induction period of oxo-biodegradable plastic film, the number of soil fungi decreased and the number of bacterium and actinobacteria increased, so the prolongation of the induction period of oxo-biodegradable plastic film can effectively maintain the balance of soil microbial populations, make the soil microbial community changed from the “fungi type” to the “bacterium type,” and reduce the occurrence of soil diseases, thus improving the soil quality. Meanwhile, it was found in this study that the application of A-SAP could significantly increase the number of soil fungi, bacterium, and actinobacteria, and this conclusion was also confirmed in the study of Bana et al. (2023), which may be due to the fact that the application of A-SAP significantly improves the soil physical properties, enhances the root vitality, and stimulates the root to secrete a large amount of inorganic and organic substances, thus creating a superior environmental condition for the activity and development of microorganisms. Under the conditions of this experiment, all the moisture-maintaining measures could increase soil microbial biomass carbon and nitrogen, but there is a significant difference in the increase rate among different treatments. Generally, straw mulching had the greatest increase, followed by ordinary transparent plastic film mulching; with the prolongation of the induction period of oxo-biodegradable plastic film, soil microbial biomass carbon and nitrogen showed an increasing change; and the result was similar to Fu et al. (2019). Whereas Song et al. (2023) concluded that plastic film mulching decreased the soil biomass carbon and nitrogen content, Li et al. (2022b) showed that the application of oxo-biodegradable plastic film had no significant effect on soil biomass carbon and nitrogen content. The difference in results may be caused by the different environment of the experimental area, farmland management measures, and crop root exudates.

### 4.5 Effect of soil moisture-maintaining measures on soil enzyme activity

Soil enzyme is often used as an important index for evaluating soil quality and soil microbial function (Ma et al., 2017). Different types of soil enzymes have different molecular compositions and structures and respond differently to a variety of factors such as external climatic conditions, soil environment, and farmland management measures. It has been confirmed that the rational use of moisture-maintaining measures can improve soil structure and provide a favorable environment for the synthesis and accumulation of soil enzymes, which, in turn, promote the improvement of enzyme activity (Chen et al., 2018). For example, ordinary plastic film, oxo-biodegradable plastic film, and straw mulching and application of A-SAP can all improve soil urease, sucrase, alkaline phosphatase, and catalase enzyme activities (Chen et al., 2021; Duanyuan et al., 2023; Yang et al., 2022b), which is basically consistent with the results of this experiment. In addition, this experiment found that the enzyme activities of urease, sucrase, and alkaline phosphatase under straw mulching have obvious effects, which may be due to the fact that straw mulching makes the shallow soil hydrothermal environment to be improved, accelerates the decomposition of straw, and then increases the soil organic matter content, which makes the original carbon and nitrogen ratio in the soil to be changed and enhances the enzymatic reaction, and stimulated the soil organisms to secrete more urease, sucrase, and alkaline phosphatase; at the same time, the surface water of soil under straw mulching increases, which increased the reductivity of catalase, and led to the decrease of catalase activity. In this study, both ordinary plastic film and oxo-biodegradable plastic film as well as application of A-SAP could increase soil cellulase activity, in which ordinary plastic film had the best effect, followed by oxo-biodegradable plastic film, which showed no significant difference in A-SAP, whereas straw mulching showed a significant inhibitory effect, with a significant reduction of 22.99% to 27.80% compared with that of CK, contrary to the results of Du et al. (2022). The reason for this may be related to the climatic environment, mulch thickness, and type of crops grown in the experimental area. It was further found that, with the prolongation of the induction period of oxo-biodegradable plastic film, the soil enzyme activity showed an incremental change, and the increase of transparent plastic film was slightly higher than that of black plastic film, so the appropriate prolongation of the induction period of transparent oxo-biodegradable plastic film can effectively improve soil enzyme activity, thus improving soil quality.

### 4.6 Effect of soil moisture-maintaining measures on yield, quality, and water–fertilizer productivity of purple garlic

The yield and quality of garlic bulbs are generally determined by varieties, climate, cultivation, management measures, etc. Reasonable moisture-maintaining measures can effectively ameliorate soil and improve soil fertility, thereby increasing crop productivity, while improving the living environment of soil microorganisms and promoting the root absorption of water and nutrients, which enables garlic to accumulate more dry matter in different development stages, thus improving bulb yield, quality, and water–fertilizer productivity (Shan et al., 2022). Li et al. (2021b) found that mulching farmland with plastic film, straw, and rice hulls can increase soil water content and enzyme activity, promote garlic plant height and bulb growth, and improve garlic yield and quality, and plastic film mulching can also improve soil temperature, so that the transparent plastic film mulching has the best effect, followed by black plastic film mulching. Xu (2015) found that plastic film mulching had a significant effect on increasing soil temperature and moisture-maintaining, and garlic plant grew vigorously, which was conducive to the accumulation and transformation of nutrients, and improved bulb quality while promoting the dry matter accumulation of the whole plant, which, in turn, increases bulb yield, whereas the garlic yield of straw mulching was significantly lower than that of plastic film mulching, and there was no significant difference in bulb quality between plastic film mulching and open field. This study further corroborates this theory. Compared with open-field planting, different moisture-maintaining measures improved garlic bulb yield and quality, of which the highest yield and best quality was achieved with 110-day transparent oxo-biodegradable plastic film, which was mainly attributed to the fact that the WL not only improved the water and fertilizer conditions but also inhibited weed growth and reduced nutrient competition in crop growth, therefore improving garlic yield and quality, whereas the yield of A-SAP increased little, which was not a strong advantage for agricultural production. Therefore, under the same irrigation and fertilization conditions, the rational selection of oxo-biodegradable plastic film type has a good water and fertilizer regulating effect, which can significantly improve the water productivity and crop yield. Therefore, in the China Hexi Corridor oasis agricultural area, where there is a serious resource-based water shortage, the application of 110-day transparent oxo-biodegradable plastic film mulching technology in garlic production can alleviate the contradiction between the crop water demand and water supply, achieve higher water–fertilizer productivity, and can increase the yield and improve the quality, which can be a feasible measure to maintain the substitution of the ordinary transparent plastic film in garlic production in the China Hexi Corridor oasis agricultural area.

## 5 Conclusion

The different moisture-maintaining measures effectively improved the soil hydrothermal environment, activated soil nutrients, changed and optimized the soil microbial growth environment, increased the number of microorganisms, and enhanced soil enzyme activities in garlic farmland in the China Hexi Corridor oasis agricultural area. The results of correlation analysis showed that soil hydrothermal, nutrients, microorganisms, and enzymes were associated with garlic yield and quality. Therefore, soil moisture-maintaining measures can improve garlic farmland soil hydrothermal environment to promote rapid microbial reproduction, increase enzyme activity, and promote nutrient decomposition, which, in turn, improves garlic yield and quality, among which 110-day transparent oxo-biodegradable plastic film mulching has the best effect on optimizing soil micro-region environment and improving garlic yield and quality, which can achieve arable land production capacity enhancement and agro-ecosystem sustainable development. However, it was still found in the test process that the current market oxo-biodegradable plastic film does not match well with the plastic film mulching needs of purple garlic, which limited the further improvement of the yield and quality of purple garlic. Therefore, the formulation of biodegradable plastic film based on ordinary polyethylene substrate blends and oxo-biodegradable additives as the main raw materials need to be further improved, especially in the regulation of degradation performance, and it is urgent to design the molecular chain structure of the material and postpone the degradation induction period to match the needs of purple garlic plastic film warming and moisture-maintaining and to achieve rapid degradation when the purple garlic enters into the mature stage. It is expected to replace the ordinary polyethylene plastic film in the production system of purple garlic in Hexi Oasis.

## Data Availability

The original contributions presented in the study are included in the article/supplementary material. Further inquiries can be directed to the corresponding author.
